# Label‐Free Clustering Analysis Platform Drives Cascaded Workflow for Scalable Production of Therapeutic Extracellular Vesicles

**DOI:** 10.1002/jev2.70333

**Published:** 2026-06-23

**Authors:** Jing Zhou, Ping Chen, Xin Chen, Xu Xiao, Haonan Di, Yunyun Hu, Yarong Zhen, Xiaomei Yan

**Affiliations:** ^1^ Discipline of Intelligent Instrument and Equipment, College of Chemistry and Chemical Engineering Xiamen University Xiamen Fujian China; ^2^ Department of Chemical Biology, MOE Key Laboratory of Spectrochemical Analysis & Instrumentation, Key Laboratory for Chemical Biology of Fujian Province, State Key Laboratory of Physical Chemistry of Solid Surfaces, College of Chemistry and Chemical Engineering Xiamen University Xiamen Fujian China; ^3^ Department of Plastic Surgery, Zhongshan Hospital of Xiamen University, School of Medicine Xiamen University Xiamen Fujian China

**Keywords:** label‐free clustering analysis algorithm, nano‐flow cytometry, therapeutic extracellular vesicles, tangential flow filtration device

## Abstract

Extracellular vesicles (EVs) have emerged as highly promising natural nanomedicines and nanocarriers, holding transformative potential for the treatment of various diseases. However, the lack of rapid and comprehensive characterization techniques for EV preparation analysis, coupled with the absence of efficient quality control methods, significantly hinders process optimization and large‐scale production. To address these challenges, we developed a label‐free clustering analysis (LFCA) platform that integrates nano‐flow cytometry for particle size distribution analysis with a clustering algorithm to deconvolute EV subpopulations and distinguish them from impurities. This platform enables the rapid quantification of EV component distribution and composition within 5 min using minimal sample input. Leveraging the high‐throughput capabilities of LFCA, we established a cascaded workflow incorporating a microcarrier‐based 3D culture system, a custom tangential flow filtration device, and multimodal size exclusion chromatography for EV preparation from adipose mesenchymal stem cells. This approach achieves a 4‐fold increase in EV yield compared to ultracentrifugation while maintaining comparable purity and preserving EV integrity. Critically, the resulting EVs exhibited enhanced functional potency in pro‐angiogenic and anti‐inflammatory assays, confirming the clinical relevance of our optimized production system. These advancements provide a scalable solution for EV production, paving the way for clinical applications.

## 1 Introduction

Extracellular vesicles (EVs) derived from mesenchymal stem cells (MSC‐EVs) are increasingly recognized as potent nanomedicines and drug‐delivery carriers, valued for their biocompatibility, ability to traverse biological barriers and robust immunomodulatory functions (Buzas 2023; Cheng et al. 2022; El Andaloussi et al. 2013; Herrmann et al. 2021; Kalluri and LeBleu et al. 2020). Encapsulating the inherent therapeutic properties of their parent MSCs, such as tissue repair and regeneration, MSC‐EVs are prime candidates for advanced cell‐free therapeutic applications (Bertolino et al. 2023; Kou et al. 2022; Tavasolian et al. 2023; Zhang and Cheng 2023). Despite their potential, clinical deployment is hampered by challenges in the scalable production of high‐purity EVs (Apostolou et al. 2023; Manno et al. 2024). Current EV isolation protocols typically involve harvesting cell culture medium followed by established separation techniques, including ultracentrifugation (Crescitelli et al. 2021; Zhang et al. 2023), immunoaffinity precipitation (Boriachek et al. 2019; Zhang et al. 2019), polymer precipitation (Liu, Zong, et al. 2022; Liu et al. 2021), flow field‐flow fractionation (Preußer et al. 2022; Zhang and Lyden 2019), anion exchange chromatography (Fang et al. 2020; Pirolli et al. 2023), size exclusion chromatography (SEC) (Guo et al. 2021; Wu et al. 2023; Zimmerman et al. 2024), microfluidic separation (Meng et al. 2023; Zhang et al. 2022) and microchip‐based methods (Chen et al. 2021; Li et al. 2024; Li, Liu, Cheng, et al. 2023). Among these, tangential flow filtration (TFF) using hollow fibre filters stands out as the preferred method for industrial‐scale production due to its scalability and efficiency. TFF employs a dynamic cross‐flow mechanism, where the liquid flows parallel to the filter membrane, effectively removing small impurities while minimizing membrane fouling and preserving EV integrity, a critical advantage for large‐scale EV operations (Busatto et al. 2018; Chernyshev et al. 2023; Hendrix et al. 2023; Romann et al. 2024; Wolf et al. 2022; Zeng et al. 2022). However, TFF alone is insufficient to produce high‐purity EVs, necessitating the integration of orthogonal separation strategies to further enhance EV purity and mitigate immunogenic risks associated with non‐EV particles (Carter et al. 2024; Iannotta et al. 2023; Keysberg et al. 2023; Kistenmacher et al. 2024; McNamara et al. 2018; Nguyen et al. 2024; Vo et al. 2024; Zhang et al. 2021). Nevertheless, the integration of multiple separation techniques often compromises process efficiency, highlighting the need for rapid and precise characterization methods to optimize production workflows. Accurate assessment of EV size, yield and purity is essential for refining production processes and ensuring the consistency of therapeutic preparations.

Given the considerable size heterogeneity of EVs, with most particles ranging from 40 to 200 nm, the development of highly sensitive and rapid single‐particle characterization techniques is crucial for advancing EV research and therapeutics. High sensitivity flow cytometry or nano‐flow cytometry (nFCM), initially developed in our laboratory, enables high‐resolution, multiparameter analysis of individual EVs as small as 40 nm, achieving sizing accuracy comparable to cryo‐TEM (Tian et al. 2018; Tian et al. 2022; Yang et al. 2009; Zhu et al. 2014). Its superior performance has been independently validated by multiple laboratories and has led to its widespread adoption in the research community (Li, Li, Li, et al. 2023; Li, Li, Ye, et al. 2023; Zhou et al. 2024). In particular, a higher proportion of particle size larger than 80 nm has been shown to correlate with greater EV purity, highlighting the strong relationship between EV purity and particle size distribution (Tian, Gong, et al. 2020). Since most separation techniques are size‐dependent, establishing a high‐throughput and precise platform that can elucidate the compositional heterogeneity within EV preparations based on size distribution analysis is critical for optimizing production efficiency to ensure therapeutic consistency and efficacy.

In this study, we address the limitations of conventional size distribution analysis methods, especially their inability to resolve internal particle clusters or distinguish between EVs and co‐isolated contaminants by developing a novel label‐free clustering analysis (LFCA) platform. This platform integrates nFCM for particle size distribution analysis with an advanced clustering algorithm, enabling rapid and accurate quantification of diverse components within EV preparations, including EVs and small protein contaminants, in just 5 min using minimal sample input (2 × 10^8^ particles/mL, 20 µL). We applied the LFCA platform to characterize adipose‐derived mesenchymal stem cell (ADSC)‐derived EVs, which are renowned for their significant therapeutic potential but are hindered by slow cell growth and low EV secretion rates (Garcia‐Contreras and Thakor 2021; Kulaj et al. 2023; Sanz‐Ros et al. 2022; Tian, Ticer, et al. 2020; Wu et al. 2023). Our results demonstrate the platform's exceptional capability in assessing EV preparations and optimizing production workflows. Notably, the LFCA platform reveals that a cascaded production process, incorporating a microcarrier‐based 3D culture system, a customized TFF device, and multimodal size exclusion chromatography (mSEC), significantly enhances both EV yield and purity. This integrated approach achieves approximately 90% purity and a yield 4‐fold higher than that of traditional ultracentrifugation, with the resulting EVs exhibiting enhanced functional potency in pro‐angiogenic and anti‐inflammatory assays, providing a scalable and efficient solution for advancing the clinical applications of ADSC‐EVs.

## 2 Materials and Methods

### 2.1 MSC Cultivation

Primary ADSC cells were revived and maintained in complete medium (DMEM/F12 supplemented with 10% EV‐depleted fetal bovine serum [FBS], 20 ng/mL bFGF and 1% P/S) and expanded to passage 4 for experimentation. In 2D culture, ADSCs proliferated rapidly in the early stages. In contrast, the 3D‐M culture system required an adaptation period, necessitating an initial seeding density 2.5 times higher than in the 2D system. The 3D‐C culture system exhibited minimal proliferation, requiring a seeding density 5 times higher than that in the 2D system, following the FiberCell operational manual. For 2D culture, 2 × 10^7^ ADSCs were seeded into six 150 mm culture dishes, each containing 20 mL of complete medium. Supernatant was collected every 3 days, yielding 108 mL after evaporation for subsequent experiments. In the 3D‐M culture system, 5 × 10^7^ ADSCs and 240 mg of microcarriers were added to 120 mL of complete medium in a 250 mL sterile spinner flask (miniSPIN‐SSF250, CytoNiche Biotech, China). The flask was placed on a 3D FloTrix mini‐SPIN system inside a 37°C, 5% CO_2_ incubator and agitated at 20 rpm for 5 min every 30 min for 24 h, followed by continuous stirring at 35 rpm. Cell viability was assessed using calcein AM/PI staining, with green fluorescence indicating live cells and red fluorescence indicating dead cells. Supernatant was collected every 3 days once the cell density exceeded 80%. Umbilical cord mesenchymal stem cells (UC‐MSCs) were cultured in the 3D‐M system under an identical protocol​ as those established for ADSCs, including seeding density, microcarrier concentration, agitation parameters and culture medium composition. This standard approach ensured consistent adaptation and comparable growth kinetics between UC‐MSCs and ADSCs within the 3D‐M environment. For the 3D‐C culture system, ADSCs were cultured in a hollow fibre cartridge‐based system (C2011, FiberCell Systems Inc.), which included a duet pump, oxygenator, hollow fibre cartridge (200 µm diameter fibres), culture medium bottle and connecting tubes. The hollow fibres had a 20 kDa molecular weight cutoff (MWCO) and a total surface area of 3000 cm^2^ in a 20 mL volume. Before cell seeding, the internal and external hollow fibre spaces were washed sequentially with 1% P/S‐PBS, DMEM/F12, and complete medium for 24 h. 1 × 10^8^ ADSCs were seeded into the external space, while 100 mL of complete medium flowed through the internal space at 25 (about 100 ∼ 110 mL/min). The media composition remained consistent with the 2D culture system, and glucose consumption was monitored every 3 days as an indicator of cell activity.

### 2.2 TFF Separation

Freshly prepared CM was transferred to sterile tubes (602052, NEST) and filtered through a 0.8 µm sterile filter (SFMPES‐8033, Cobetter) to remove large debris. EVs were then separated and enriched using a custom‐designed TFF system. Initially, 120 mL of CM was concentrated to ∼60 mL through circulating filtration. The system was then switched to washing‐filtration mode to remove non‐EV particles, continuing until the sample appeared colourless. During which, small molecules (e.g. glucose and nucleic acids) smaller than the membrane pore size were removed with the filtrate. A final concentration reduced the sample volume to ∼10 mL, yielding the retentate. To recover EVs adhering to the system, 30 mL of PBS was used to rinse the system, and the remaining preparation was concentrated to ∼10 mL, yielding the W1 sample. The rinse step was repeated, generating a W2 sample. EV concentration and size distribution in the retentate, W1 and W2, were measured using the LFCA platform. Since particle adhesion is related to the surface area (SA) of the system, the lengths, diameters, and filtering areas of the components were measured, resulting in a SA of 200 cm^2^. The number of adhered particles per unit area in W1 and W2 was analyzed. The feed flow rate was maintained at 100 mL/min, with a maximum transmembrane pressure (TMP) of 3 psi to preserve EV lipid membrane integrity. TMP was calculated as:

$$\mathrm{TMP}=\frac{P_{1}+P_{2}}{2}-P_{3}$$

where *P*_1_ is the fluid pressure at the filter cartridge inlet, *P*_2_ is the fluid pressure at the retentate outlet and *P*_3_ is the fluid pressure at the permeate outlet.

### 2.3 Chromatographic Purification

For chromatographic purification, 1 mL of TFF‐EV preparation (concentration: 5 × 10^10^ to 5 × 10^11^ particles/mL) was loaded onto the chromatography column. The initial PBS flowing‐through was collected as fraction 1 (F1). During elution, 0.5 mL of PBS was added sequentially, with equal volumes of eluate collected and denoted as F2 to F24. For SEC, F1 contained PBS retained outside and within the gel pores, representing the ∼ 1 mL void volume. Fractions were pooled as follows: F6‐F8 were combined as F6, F9‐F12 were combined as F7, and F12‐F24 were combined as F8. For mSEC, the void volume F1 was ∼2 mL, and F8‐F24 were combined as F8. For AEC, after sample loading, weakly bound nanoparticles were removed with 12 mL of wash buffer, followed by 24 cycles of 0.5 mL elution buffer. Fractions were pooled as follows: F1‐F9 were combined as F1, fractions F10 to F14 were renamed F2 to F6, F15‐F16 were combined as F7, and F17‐F24 were combined as F8. EV‐enriched fractions were collected for further analysis. The equilibration buffer was sodium phosphate buffer (PB) with 50 mM NaCl, the wash buffer contained PB with 100 mM NaCl, and the elution buffer consisted of PB with 500 mM NaCl.

### 2.4 nFCM Analysis

nFCM analysis was performed using a Flow NanoAnalyzer model U30 (NanoFCM Inc., Xiamen, China) equipped with a 488 nm and a 637 nm laser. Optical alignment was first performed using 200 nm polystyrene (PS) beads. After alignment, particle concentration was calibrated using 250 nm silica quality control beads (QS2503, NanoFCM), with a defined concentration of 2.08 × 10^8^ particles/mL. To establish a calibration curve for side scattering (SSC) intensity‐to‐size conversion, the SiNPs Cocktail (S16M‐Exo, NanoFCM), consisting of 68, 91, 113 and 155 nm SiNPs, was used as a size reference material. To define background noise levels, freshly filtered (0.22 µm) 1× PBS was analyzed, and its signal was subtracted from all measurements. Each distribution histogram or dot plot was generated from data collected over 1 min at a sample pressure of 1 kPa. EV samples were diluted in filtered 1× PBS to achieve an optimal event range of 4000–8000 particles. Particle concentration and size distribution were calculated using the NF Profession V2.0 software (NanoFCM).

### 2.5 Implementation of the LFCA Platform

The particle size distribution histogram of EV preparation obtained from nFCM was processed using the LFCA algorithm. The number of potential subpopulations (*N*1) was preliminarily estimated based on local extrema, and the search range for the number of Gaussian components was set as K∈[N1,N1+2] to minimize subpopulation omission caused by noise interference. The dataset was decomposed using the GMM‐EM iterative approach, yielding the original histogram, probability density curves of each component, and an overall fitted curve. The biological significance of the Gaussian components, such as vesicles and protein aggregates, was validated through TEM morphological characterization and Triton X‐100 membrane lysis assays. TEM analysis was performed to examine particle morphology, including membrane integrity and size uniformity, while Triton X‐100 lysis was used to differentiate lipid membrane structures from non‐membranous aggregates. Finally, the clustering results were integrated with physicochemical parameters to construct characteristic distribution profiles of EVs and co‐isolated contaminants.

### 2.6 Assay of Pro‐Angiogenic, Scratch Repair and Anti‐Inflammatory Capacities of EVs

To assess the pro‐angiogenic capacity of EVs, a tube formation assay was performed using human umbilical vein endothelial cells (HUVECs). Pipettes, 24‐well plates, and Matrigel were pre‐cooled to −20°C. Thawed Matrigel was gently mixed on ice, and 20 µL of 2:1 dilution was aliquoted into each of the pre‐cooled 48‐well plate using chilled pipettes. The plate was incubated overnight at 4°C to facilitate gelation and self‐levelling of the Matrigel, followed by a 30‐min incubation at 37°C to promote solidification. HUVECs at 80% confluence were digested, resuspended in DMEM supplemented with 1% EV‐depleted FBS, and counted via trypan blue dilution (1:1) to ensure > 90% cell viability. A working solution containing EVs (1 × 10^9^ particles/mL) and HUVECs (3 × 10^5^ cells/mL) was prepared in DMEM with 1% EV‐depleted FBS. Subsequently, 500 µL of the cell‐EV suspension was carefully added to each Matrigel‐coated well to avoid bubble formation. The plate was cultured at 37°C for 4 h, after which tube formation was visualized and imaged using an Olympus IX73 microscope. The total vessel length was quantified from the acquired images.

The migratory capacity of cells treated with EVs was evaluated using a scratch wound healing assay. Cells were resuspended to a density of 3 × 10^5^ cells/mL, and 500 µL of the suspension (approximately 1.5 × 10^5^ cells/well) was seeded into 24‐well plates. The cells were cultured for 12–24 h until they reached > 90% confluence. A uniform scratch was created in the monolayer using a sterile 10 µL pipette tip. The wells were gently washed twice with PBS to remove detached cells. A working solution of EVs at a concentration of 1 × 10^9^ particles/mL was prepared in DMEM containing with 1% EV‐depleted FBS. The original culture medium was replaced with the EV working solution. After 12 h of incubation, the scratch morphology was observed and imaged. The degree of wound closure was assessed by calculating the scratch area ratio compared to the initial wound area at 0 h.

The anti‐inflammatory effects of EVs were investigated using BV2 microglial cells. When BV2 cells reached 80% confluence, they were seeded at a density of 2 × 10^5^ cells/mL (200 µL per well) into the centre of confocal dishes and cultured for 12–24 h until the confluence exceeded 85%. The culture medium was then replaced with one of the following treatments: (1) blank control group (fresh medium only), (2) LPS group (medium containing 100 ng/mL of lipopolysaccharide, LPS), (3) UC‐EVs group (100 ng/mL LPS plus 1 × 10^9^ particles/mL UC‐EVs), (4) TFF‐EVs group (100 ng/mL LPS plus 1 × 10^9^ particles/mL TFF‐EVs), or (5) TFF‐mSEC‐EVs group (100 ng/mL LPS plus 1 × 10^9^ particles/mL TFF‐mSEC‐EVs). After 24 h of incubation, the culture medium was collected for enzyme‐linked immunosorbent assay (ELISA) to quantify the secretion levels of tumour necrosis factor‐alpha (TNF‐α; ABclonal, RK00027‐96‐T) and interleukin‐6 (IL‐6; ABclonal, RK00008‐96‐T).

For immunofluorescence staining, the cells were fixed with 4% paraformaldehyde (Putai Biotech, P0099‐500 mL), washed with PBS (Procell, PB180327), and subsequently permeabilized and blocked with a solution containing 5% goat serum (Bioss, BD08134382) and 0.1% Triton X‐100 (Sigma‐Aldrich, SL09531). The primary antibody against inducible nitric oxide synthase (iNOS; Abcam, ab178945) was diluted 1:400 in PBS containing 0.01% Triton X‐100 and 2% goat serum. Then, 200 µL of the antibody solution was added to each well and incubated at 4°C for 12 h. After three washes with PBS, the cells were incubated with 200 µL of Alexa Fluor 488‐conjugated secondary antibody (Proteintech, SA00013‐2) at a 1:100 dilution for 1–1.5 h at room temperature. Following another three washes with PBS, the nuclei were counterstained with 200 µL of Hoechst 33342 (Yeasen, 40732ES03) working solution (1:1000 dilution) for 15 min at room temperature in the dark. After a final series of PBS washes, fluorescence imaging was performed using a high‐resolution confocal fluorescence microscope (Leica SP8‐STED 3X). The fluorescence intensity of iNOS staining was quantified to assess the anti‐inflammatory efficacy of the EVs.

### 2.7 Statistical Analysis

All statistical analyses were conducted using Origin 9.1 and GraphPad Prism 9.0. Data are presented as the mean ± SD. Significant differences between groups were determined using one‐way or two‐way ANOVA, as appropriate, followed by Tukey's post hoc test. Statistical significance was defined as *P* > 0.05 (ns), *P* ≤ 0.05 (*), *P* ≤ 0.01 (**), *P* ≤ 0.001 (***), *p* ≤ 0.0001 (****). Schematic diagrams were created using Adobe Illustrator 2021, and the specialized TFF components were designed using SOLIDWORKS 2018.

## 3 Results

### 3.1 Working Principle of the LFCA Platform

**FIGURE 1:**
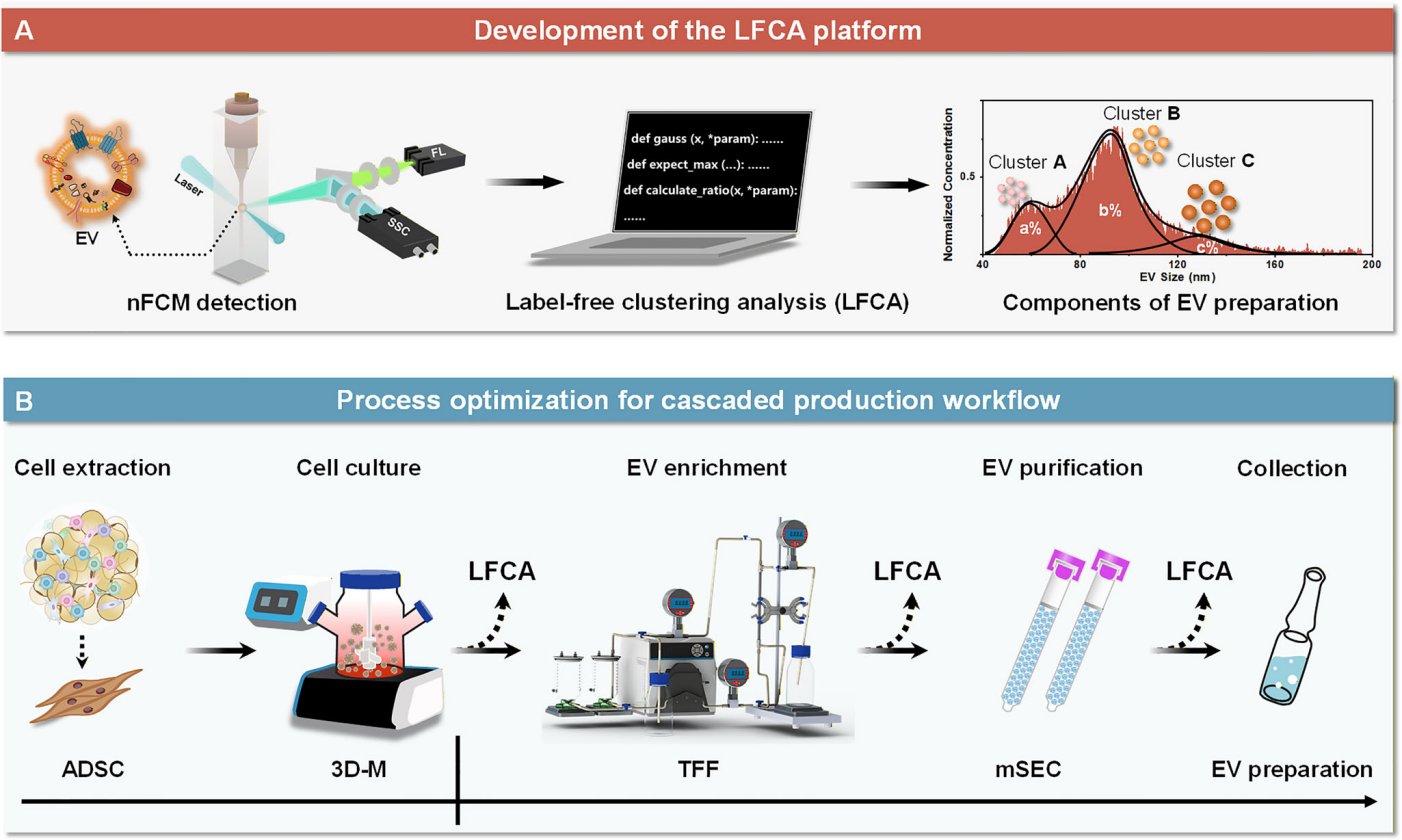
Schematic illustration of the LFCA platform and its application in optimizing the cascaded production workflow for ADSC‐derived EVs. (A) Overview of the LFCA platform, depicting its integration of nFCM to measure the size distribution of nanoparticles in EV preparations and the application of label‐free clustering analysis using the GMM and the EM algorithm. (B) Workflow diagram of the cascaded production process for ADSC‐derived EVs, highlighting the application of the LFCA platform at various stages, including 3D culture, TFF for separation and enrichment and mSEC for purification.

Figure 1A presents the schematic of the LFCA platform's working principle. This platform integrates nFCM to measure the size distribution of EV preparations, which include both EVs and impurities, and a clustering algorithm to resolve different nanoparticle populations. The high‐resolution particle size distribution data obtained from nFCM exhibits inherent sparsity, challenging traditional methods such as polynomial fitting or extremum analysis to estimate Gaussian distribution parameters accurately (Malle et al. 2024; Tian, Gong, et al. 2020; Tian et al. 2018). To overcome these challenges, the clustering algorithm employs the Gaussian Mixture Model (GMM) and the Expectation‐Maximization (EM) algorithms. GMM, a widely used probabilistic model in gene expression pattern analysis (Ficklin et al. 2017; Liu, Kalugin, et al. 2022), disease‐associated biomarker analysis (Hossain and Balagopal 2023), biological image processing (Li et al. 2017) and single‐cell sequencing (Wang et al. 2021), assumes that data comprises multiple subpopulations, each following a Gaussian distribution (McLachlan and Chang 2004). By utilizing weighted probability density functions, GMM performs soft clustering to decompose size distribution data into Gaussian‐distributed clusters, enabling precise size‐based resolution of mixed nanoparticles without the need for pure standards. The EM algorithm (Redner and Walker 1984), a classical unsupervised machine learning approach, iteratively refines key parameters such as mean, variance, and cluster probabilities within the GMM framework.

Leveraging these methodologies, we developed a Python‐based algorithm that enhances the LFCA platform's clustering capabilities. Specifically, the LFCA algorithm models the probability density function of the size distribution data using GMM and optimizes the mean, variance, and mixture weight parameters iteratively via the EM algorithm. The workflow begins with data normalization and parameter initialization, followed by the E‐step, which computes the posterior probability distribution, and the M‐step, which iteratively updates the parameters until convergence. The goodness of fit is assessed using the residual sum of squares (RSS), ensuring accurate model performance. A detailed mathematical derivation of the LFCA algorithm is provided in the Supplementary Note. The algorithm is implemented in Python 3.7, with core dependencies including numpy, scipy, pandas and matplotlib.

Figure 1B illustrates the application of the LFCA platform in a cascaded production workflow for therapeutic EVs derived from ADSCs, incorporating a microcarrier‐based 3D culture system, a customized TFF device, and an mSEC column. At each step, the LFCA platform analyses EV preparations to assess changes in clustering components, facilitating the optimization of production conditions and parameters. This comprehensive analysis enables the establishment of an optimal EV production workflow.

To validate the clustering capabilities of the LFCA platform, we analyzed mixtures containing 78 nm fluorescent silica nanoparticles (FL‐SiNPs) at varying proportions, along with non‐fluorescent SiNPs of different sizes (69, 90, 113 and 155 nm). As depicted in Figure S1A, gating analysis was employed to accurately quantify the proportion of 78 nm FL‐SiNPs in each mixture, specifically by utilizing the characteristic fluorescence signal of the 78 nm FL‐SiNPs. Concurrently, the LFCA platform conducted clustering analyses on the size distribution histograms (Figure S2B) and automatically calculated the proportions of 78 nm FL‐SiNPs. A comparative analysis of the results from the gating method and the LFCA platform revealed a strong linear correlation, as illustrated in Figure S2C, with a regression line slope of 0.9943 and an *R* ^2^ value of 0.9967. These findings underscore the LFCA platform's high precision and robustness in resolving size distributions and effectively clustering mixed nanoparticles, despite the partial overlap between the size distributions of 78 nm FL‐SiNPs and those of 69 and 90 nm SiNPs.

### 3.2 Construction of Three ADSC Culture Systems and Analysis of ADSC‐Derived EVs

**FIGURE 2:**
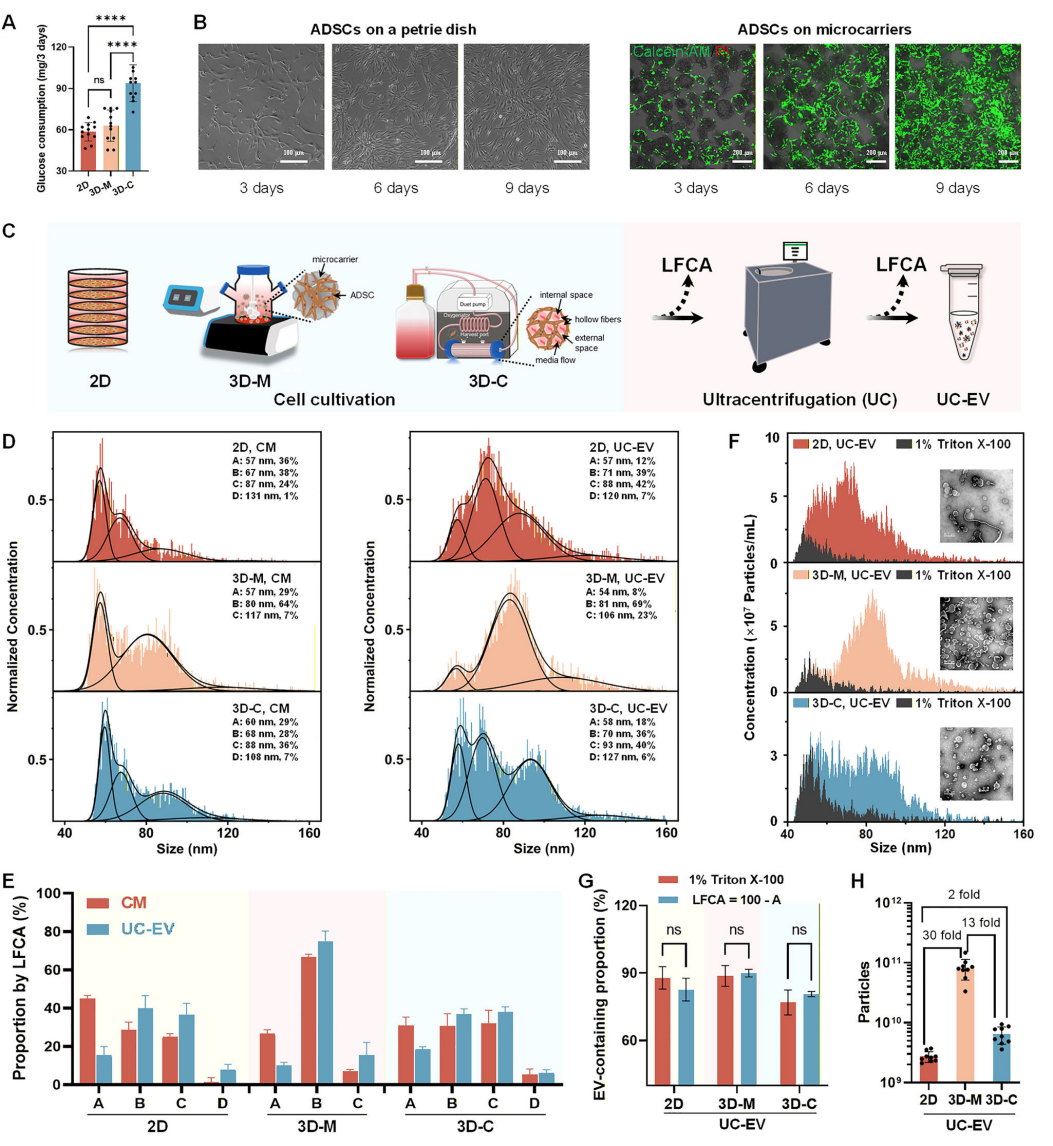
Construction of three ADSC culture systems and analysis of derived EVs. (A) Glucose consumption profiles in 2D, 3D‐M and 3D‐C culture systems measured over a period of 3 days (*n* = 11; independent technical replicates, mean ± SD). (B) Optical microscopy images of ADSCs on days 3, 6 and 9 within the 2D (scale bar: 100 µm) and 3D‐M (scale bar: 200 µm) systems. (C) Schematic of the workflow for nanoparticle clustering analysis across different culture systems using the LFCA platform. (D) Clustering analysis of CM and UC‐EVs from the 2D, 3D‐M and 3D‐C culture systems. (E) Proportional changes in clustered components within CM and UC‐EV preparations across the culture systems. (F) Size distribution analysis of UC‐EVs before and after Triton X‐100 lysis; inset shows corresponding TEM images (scale bar: 0.5 µm). (G) Statistical analysis of cluster proportions obtained via Triton X‐100 lysis and LFCA platform analysis (*n* = 3; independent technical replicates, mean ± SD). (H) Comparison of UC‐EV yields per mL per 72 h from the 2D, 3D‐M and 3D‐C systems (*n* = 9; independent technical replicates; mean ± SD).

ADSCs were isolated from surplus human adipose tissue and confirmed through flow cytometry and trilineage differentiation assays, meeting International Society for Cellular Therapy (ISCT) standards (Figures S2–S4). The cells were cultured in three systems: two‐dimensional (2D), microcarrier‐based three‐dimensional (3D‐M), and hollow fibre cartridge‐based three‐dimensional (3D‐C). The 3D‐C system exhibited significantly higher glucose consumption over 3 days (94 mg) compared to the 2D (59 mg) and 3D‐M (63 mg) systems (Figure 2A). Optical microscopy revealed that ADSCs in both 2D and 3D‐M systems achieved over 80% confluence by day 9, indicating robust proliferation (Figure 2B).

For EV production analysis, nFCM assessed the size distribution, yield, and composition of nanoparticles in conditioned medium (CM) and ultracentrifugation‐prepared EVs (UC‐EVs). The median nanoparticle sizes in CM were 63, 70 and 65 nm for the 2D, 3D‐M and 3D‐C systems, respectively, with concentrations of 1.5 × 10^9^, 4.2 × 10^9^ and 7.7 × 10^9^ particles/mL, indicating a significant increase in the 3D systems compared to the 2D system (Figure S5A,B). Upon UC purification, the median sizes of nanoparticles in UC‐EVs were 75, 87 and 76 nm for the 2D, 3D‐M and 3D‐C systems, respectively (Figure S5C,D), with similar median sizes (∼75 nm) noted between the 2D and 3D‐C systems, despite significant differences in their particle‐to‐protein ratios (Figure S5E). Further analysis using the LFCA platform revealed four clusters based on median size in CM samples from the 2D and 3D‐C systems, and three clusters in those from the 3D‐M system (Figure 2C,D). The 3D‐M system notably increased the proportion of clusters larger than 80 nm in both CM and UC‐EVs. Cluster analysis before and after UC highlighted significant compositional changes; UC substantially reduced the proportion of Cluster A while enriching Clusters B, C and D, suggesting Cluster A mainly consists of non‐EV components (Figure 2D,E).

To further investigate Cluster A, Triton X‐100 was used to lyse UC‐EVs (Hergenreider et al. 2012). Post‐lysis analysis revealed that the unlysed nanoparticles in UC‐EVs from the 2D, 3D‐M and 3D‐C systems predominantly fell within the size range of Cluster A, confirming that it primarily consists of non‐EVs lacking lipid membrane structures (Figure 2F). Transmission electron microscopy (TEM) of UC‐EVs provided further characterization, showing that non‐EVs lacked the typical cup‐shaped morphology and displayed smaller particle sizes (Figure 2F, inset). These findings suggest that subtracting the proportion of Cluster A from the total nanoparticle count using the LFCA platform allows for accurate quantification of EV purity. The purities of UC‐EVs from the 2D, 3D‐M and 3D‐C system were determined to be 88%, 89% and 77%, respectively using Triton X‐100 lysis, closely aligning with values obtained via the LFCA platform (83%, 90% and 81%) (Figure 2G). This consistency between the two quantification methods underscores the LFCA platform's utility in revealing structural clustering changes across CM and UC‐EVs, offering valuable insights for optimizing cell culture and EV isolation techniques.

Notably, the 3D‐M system markedly increased ADSC‐derived EV secretion, yielding approximately 30‐fold and 13‐fold higher yields compared to the 2D and 3D‐C systems, respectively (Figure 2H). Specifically, UC yielded 2.7 × 10^9^, 8.3 × 10^10^ and 6.4 × 10^9^ EVs per mL per 72 h from the 2D, 3D‐M and 3D‐C systems, respectively, under identical cell culture durations and medium volumes. Despite higher glucose consumption, the 3D‐C system's EV yield was constrained by hypoxia due to insufficient oxygen exchange through the latex tube, limiting aerobic metabolism (Tan et al. 2024). In contrast, the 3D‐M system, equipped with a breathable membrane cap and continuous magnetic stirring, ensured efficient gas exchange, nutrient absorption and EV production. Culture conditions also influenced the quality of ADSC‐derived EVs. The particle‐to‐protein ratios for UC‐EVs from the 2D, 3D‐M and 3D‐C systems were 1.4 × 10^8^, 1.5 × 10^8^ and 6.3 × 10^7^ particles/µg protein, respectively (Figure S5E). Phenotypic analysis showed enhanced expression of key markers such as CD9, CD81, CD73 and CD90 in 3D‐M UC‐EVs compared to their 2D‐ and 3D‐C counterparts, with CD73 expression significantly higher in the 3D‐M UC‐EVs (Figures S6–S9). Given CD73's known role in immunomodulation and tissue regeneration (Hou et al. 2024; Li et al. 2021). its elevated presence emphasized its potential as a functional contributor and biomarker for therapeutic applications of ADSC‐derived EVs. Moreover, the significantly higher CD90 fluorescence in 3D‐M UC‐EVs suggests an increased marker density per EV, reinforcing the enhanced phenotypic and functional profile of these EVs. These findings highlight the critical impact of cell culture conditions on the yield and functional profiles of ADSC‐derived EVs.

### 3.3 Design Principle and Application of the Custom‐Built TFF System for EV Separation

**FIGURE 3:**
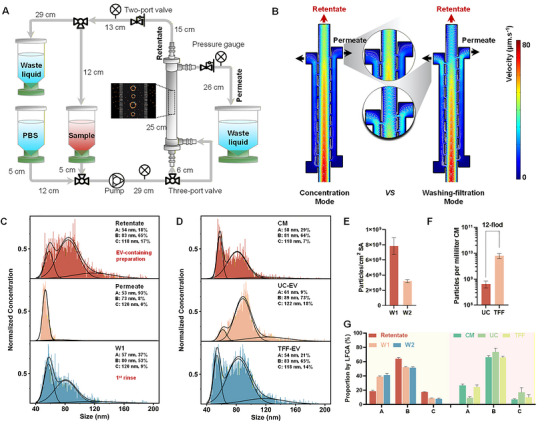
Application of the LFCA platform in the evaluation of TFF separation process. (A) Schematic of the flow path design within the custom‐built TFF device. (B) Results from CFD simulations illustrating the dynamics within the TFF device under Concentration Mode and Washing‐filtration Mode. (C) Clustering analysis of nanoparticles obtained from the TFF retentate, permeate, and the first rinse (W1) of the TFF device's inner surface. (D) Clustering analysis of CM, UC‐EVs and TFF‐EVs. (E) Comparison analysis of particle concentrations per unit area for nanoparticles obtained from the first rinse (W1) and second rinse (W2) of the TFF device's inner surface. (F) Comparison of nanoparticle yields between UC and TFF methods. (G) Comparison of the proportions of Clusters A, B and C in the retentate as well as in the first rinse and second rinse collected during the TFF process. Additionally, the proportions of these clusters are presented for the CM, UC‐EVs and TFF‐EVs (*n* = 3; independent technical replicates; mean ± SD).

To address the scalability limitation of UC for EV production, we developed a custom‐built TFF system, illustrated in Figure S10. This system utilized 3.2 mm diameter silicone tubing configured to establish a continuous flow, optimizing the separation process (Figure 3A). To further optimize the separation process, computational fluid dynamics (CFD) simulations were employed to model particle flow dynamics within the hollow fibre filter, comparing Washing‐filtration Mode and Concentration Mode (Figure S11A,B). In the Washing‐filtration Mode, an increased liquid velocity at the filtrate outlet facilitates the transit of smaller particles through the membrane pores, thereby enhancing permeation and improving separation efficiency (Figure 3B). Therefore, the operation workflow of the TFF system includes Primary Concentration, Washing‐filtration and Secondary Concentration (Figure S12), complemented by both Regular and Chemical Cleaning steps to restore membrane water flux (Figures S13–S14). The system incorporates multifaceted flow strategies, Washing‐filtration, Concentration, Cleaning and Back‐flushing, offering substantial adaptability for customization based on specific operational needs (Figure S11).

The performance of the TFF system for EV separation and enrichment was evaluated using CM from the 3D‐M culture system, which was selected for its high EV yield. As depicted in Figure 3C, the TFF system effectively excluded clusters smaller than 60 nm. During the TFF process, some EVs may adhere to the system's piping and membrane surfaces. Cyclic rinsing with PBS was employed to recover EVs adhering to these surfaces. Sequential collections from two rinse cycles (W1 and W2) were analyzed for clustering using the LFCA platform, revealing a progressive increase in the proportion of Cluster A with each rinse (Figure S15). Quantitative analysis indicated that the number of nanoparticles per unit surface area was approximately 7.9 × 10^8^ and 3.2 × 10^8^ particles/cm^2^ for the first and second rinses, respectively, suggesting that a single rinse efficiently collects the majority of EVs (Figure 3E). Consequently, the combined retentates from the retentates of the initial run and the first rinse (W1) were collected and designated as TFF‐EVs for all subsequent experiments. Comparative analyses of CM, UC‐EVs and TFF‐EVs using the LFCA platform showed that while TFF is less effective than UC at removing Cluster A from CM, it yields significantly more nanoparticles (8.1 × 10^9^ particles/mL CM), approximately twelve times greater than UC (6.5 × 10^8^ particles/mL CM) (Figure 3D,F). A summary of clustering structures from various processing steps is presented in Figure 3G. The high yield of TFF offers a significant advantage by minimizing the loss of functional EV subpopulations; however, additional post‐processing steps are needed to enhance EV purity.

### 3.4 LFCA Analysis of TFF‐EVs Purified by Chromatographic Methods

**FIGURE 4:**
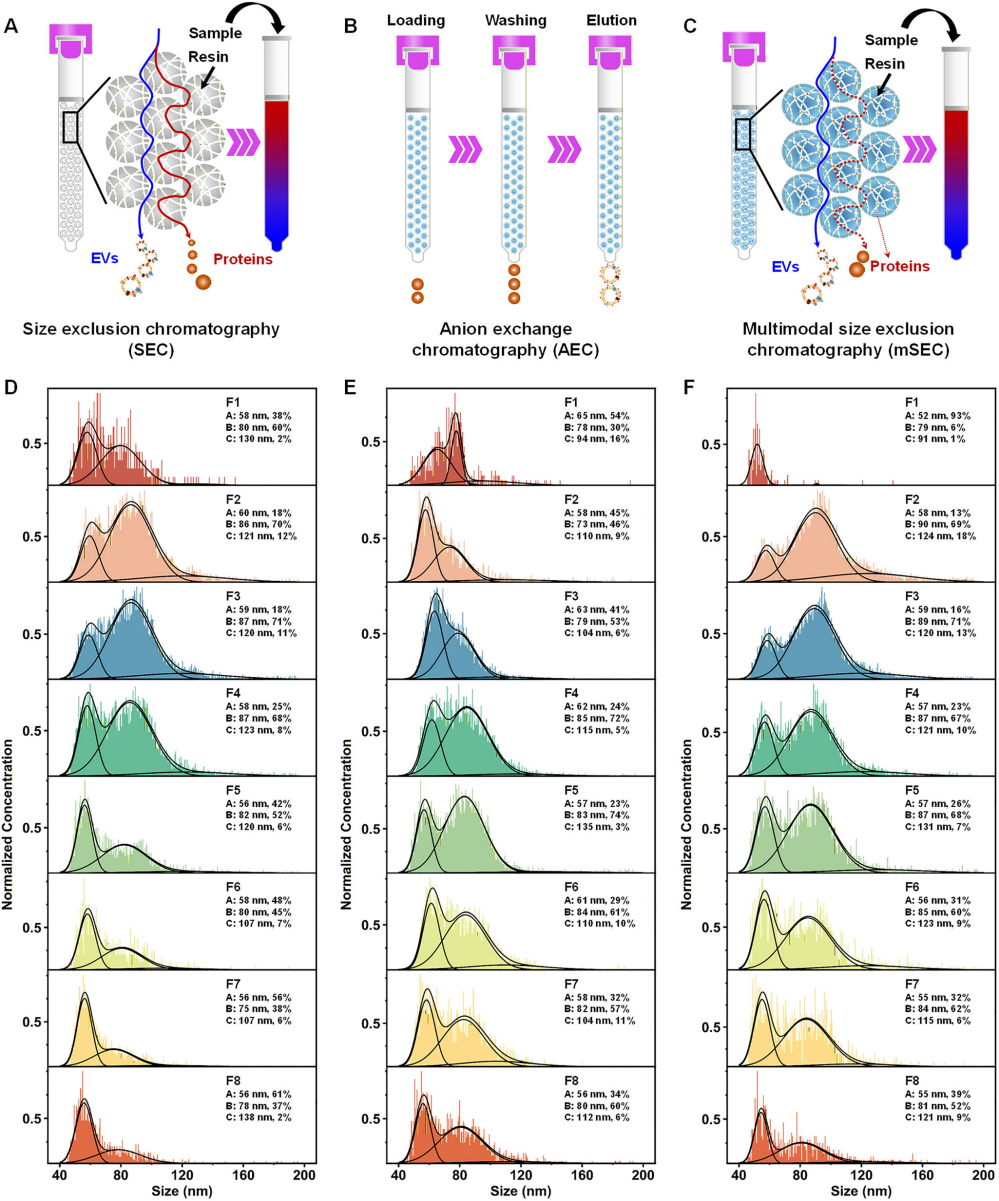
Clustering analysis of nanoparticles across chromatography fractions using the LFCA platform. (A) Schematic of the SEC process, demonstrating size‐based particle separation. (B) Overview of the AEC method, highlighting charge‐based particle sorting. (C) Depiction of the mSEC technique, combining size exclusion with hydrophobic and electrostatic interactions for enhanced purification. (D–F) Clustering analysis results for each fraction from SEC (D), AEC (E) and mSEC (F).

Figure 4 illustrates the applications of three distinct chromatographic methods for further purifying TFF‐EVs: size exclusion chromatography (SEC) using qEV 70 resin (Figure 4A), anion exchange chromatography (AEC) with Q Sepharose Fast Flow resin (Figure 4B), and multimodal size exclusion chromatography (mSEC) employing Capto Core 700 resin (Figure 4C). Each method operates through different purification mechanisms: SEC separates particles based on size, AEC utilizes electrostatic interactions to sort nanoparticles by charge, and mSEC combines size exclusion with additional hydrophobic and electrostatic interactions, improving EV retention and exclusion of non‐EV contaminants. nFCM was used to evaluate the size distribution and concentration of nanoparticles across the collected fractions, illustrating each method's effectiveness in purifying TFF‐EVs. For SEC, nanoparticles were effectively separated in fractions F2‐F4, which displayed the highest median sizes and particle concentrations, tapering off in subsequent fractions (Figure S16A). AEC results showed a significant enrichment of nanoparticles in fractions F3‐F5, where nanoparticles maintained stable median sizes but exhibited higher concentrations relative to other fractions (Figure S16B). In contrast, mSEC achieved peak separation efficiency in fractions F2‐F4, beyond which particle concentration sharply declined (Figure S16C). These data indicate the superior isolation capabilities of SEC and mSEC within the specific fractions F2‐F4, while AEC shows its highest efficacy in enriching nanoparticles within fractions F3‐F5.

LFCA clustering analysis was conducted on each fraction from SEC, AEC and mSEC to assess the compositional changes of EVs and non‐EVs. In SEC, the proportion of Cluster A significantly reduced in fractions F2 and F3 compared to that in TFF‐EVs (Figure 4D vs. Figure 3D), signifying an effective separation of non‐EVs from EVs. As the fraction number increased, there was a notable rise in the proportion of Cluster A, while Clusters B and C diminished, indicating accumulating non‐EV components in later fractions. In AEC, fractions F4 and F5 exhibited the lowest proportions of Cluster A among all analyzed AEC fractions; however, their levels remained comparable to that of TFF‐EVs (Figure 4E vs. Figure 3D). This pattern not only suggests that AEC is less effective in removing non‐EVs but also indicates the potential loss of larger EVs, as evidenced by the disappearance of Cluster C. For mSEC, the proportion of Cluster A in fraction F2 was markedly reduced to 13%, demonstrating mSEC's superior capability in separating non‐EVs from EVs (Figure 4F). Further analysis in Figure S17 reveals that mSEC more effectively retains non‐EV components across successive fractions compared to SEC, as indicated by a smaller increase in the proportion of Cluster A. This enhanced retention of Clusters A makes mSEC particularly effective for further purifying EVs. These analyses highlight the utility of LFCA in evaluating the clustering structures of EVs and provide critical insights for selecting the most effective chromatography methods to isolate high‐purity EV fractions, thus optimizing purification strategies for TFF‐EVs.

### 3.5 Utilizing the LFCA Platform for Enhancing EV Separation Through Integrated Strategies

**FIGURE 5:**
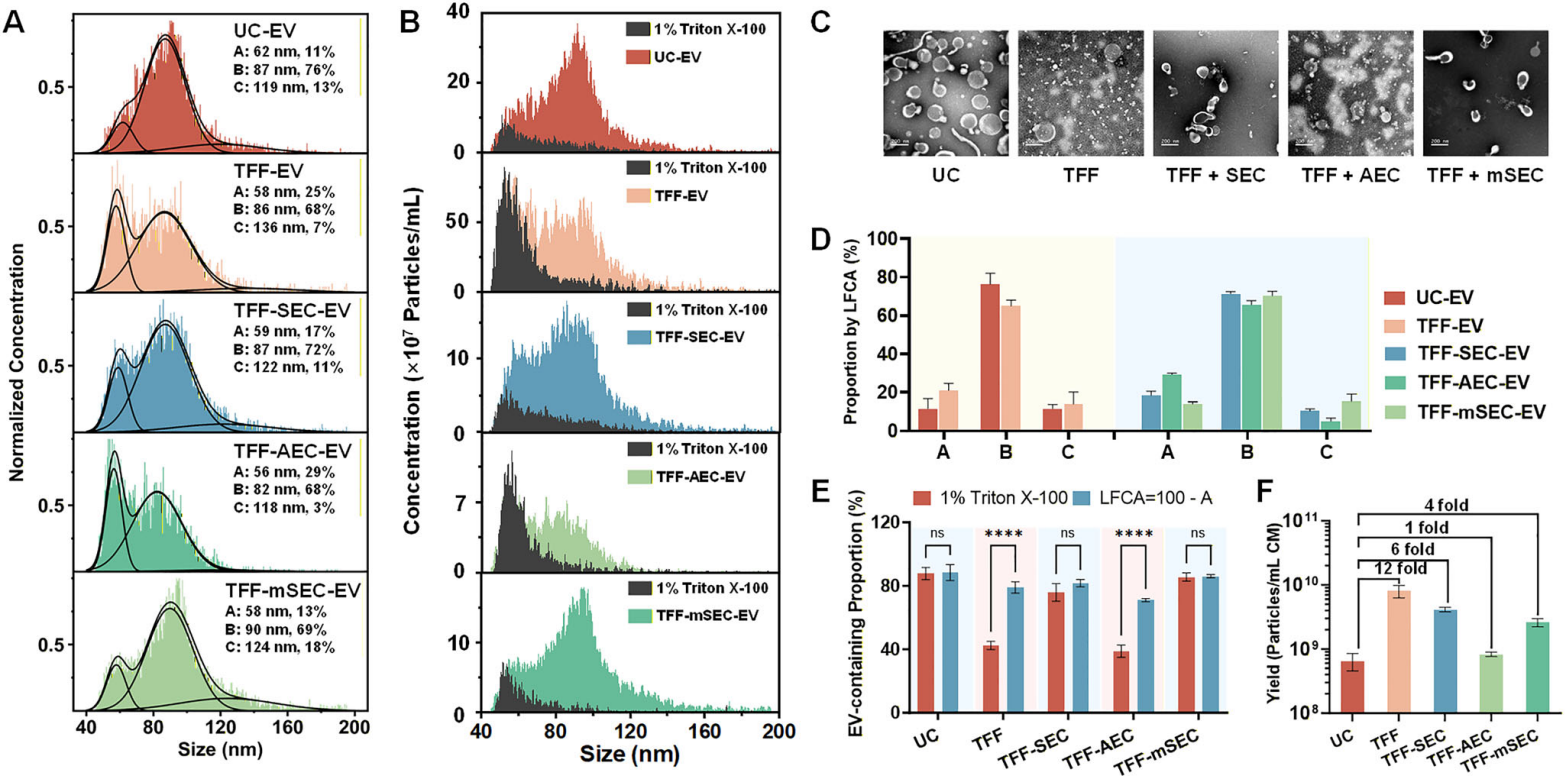
Application of the LFCA platform in single and combined separation strategies. (A) Clustering analysis comparing EV preparations obtained using UC, TFF, TFF‐SEC, TFF‐AEC and TFF‐mSEC strategies. (B) Size distribution histograms of EV preparations before and after Triton X‐100 lysis. (C) TEM images of EV preparations obtained through different separation strategies (scale bar, 200 nm). (D) Proportions of Clusters A, B and C in EV preparations obtained using different separation strategies. (E) Comparative analysis of EV purity in preparations assessed by Triton X‐100 lysis versus LFCA platform analysis. (F) Comparison of EV yield across different separation strategies (*n* = 3; independent technical replicates; mean ± SD).

Using the LFCA platform, we analyzed EV preparations pooled from selected fractions: specifically, fractions F2‐F4 for SEC and mSEC, and fractions F4‐F5 for AEC. These were denoted as TFF‐SEC‐EVs, TFF‐mSEC‐EVs and TFF‐AEC‐EVs, respectively. Compared to EVs obtained from single separation strategies (UC‐EVs and TFF‐EVs), these integrated approached demonstrated significant differences in both yield and EV purity (Figure 5A). Triton X‐100 lysis confirmed that nanoparticles primarily in Cluster A, which are predominantly composed of non‐EV particles lacking lipid membranes, remained intact and were not lysed (Figure 5B). TEM analyses revealed that non‐EVs are generally smaller than EVs. Notably, TFF‐processed EVs exhibited a more spherical and intact structure compared to those isolated via UC (Figure 5C). Statistical analysis indicated that UC and TFF‐mSEC were more effective in reducing Cluster A, outperforming TFF‐SEC. In contrast, TFF and TFF‐AEC demonstrated limited effectiveness. Notably, TFF‐AEC not only failed to eliminate Cluster A but also significantly reduced Cluster C (Figure 5D).

Purity assessments using both Triton X‐100 lysis and the LFCA platform demonstrated consistency for UC, TFF‐SEC and TFF‐mSEC. Specifically, the purities measured via Triton X ‐ 100 lysis were 88%, 82% and 86% for UC, TFF‐SEC and TFF‐mSEC, respectively. Correspondingly, the purities measured via LFCA were 88%, 86% and 86% for the same methods, respectively. However, for TFF and TFF‐AEC, the purity levels assessed by Triton X‐100 lysis were substantially lower than those measured by LFCA (42% and 39% via Triton X‐100 versus 79% and 71% via LFCA, respectively) (Figure 5E). This discrepancy may result from the lysis of non‐EV lipoproteins into smaller particles, which artificially increases the post‐lysis particle counts and thus underestimates purity. TEM further supported these findings, indicating that while UC, TFF‐mSEC and TFF‐SEC produced high‐purity EVs with minimal contamination, TFF‐AEC not only failed to improve purity but also induced EV deformation, shrinkage, and aggregation due to high‐salt conditions during processing (Figure 5C). The additional processing steps in the combined separation strategies of TFF progressively reduced nanoparticle yield. Nonetheless, compared to UC, TFF‐SEC, TFF‐AEC and TFF‐mSEC increased particle yield by 6‐fold, 1‐fold and 4‐fold, respectively (Figure 5F). Notably, mSEC was particularly effective, significantly enhancing EV yield while maintaining high purity.

### 3.6 Functional Enhancement of TFF‐mSEC‐EVs and Universality of the LFCA Platform

**FIGURE 6:**
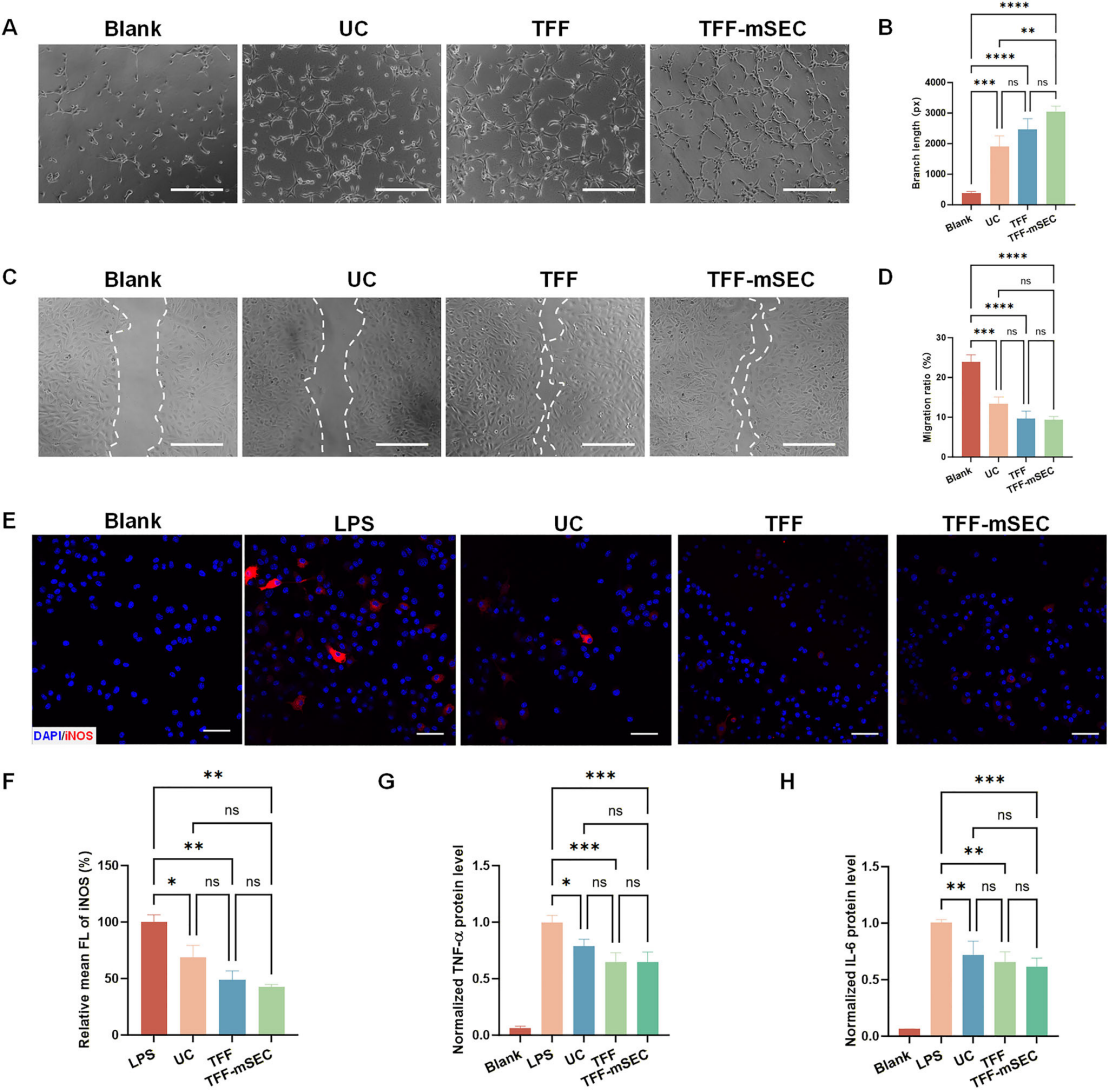
Functional validation of ADSC‐EVs reveals enhanced bioactivity of TFF‐mSEC‐EVs and superior anti‐inflammatory potency. (A) Tube formation by HUVECs following a 4‐h incubation with EVs from the indicated groups (scale bar: 100 µm). Groups: Blank (no EVs), UC‐EVs, TFF‐EVs, TFF‐SEC‐EVs. (B) Migration of HUVECs in a scratch‐wound assay at 12 h post‐scratching (scale bar: 100 µm). (C) Quantification of the relative total branch length in the tube formation assay. (D) Quantification of HUVEC migration distance at 12 h. (E) Immunofluorescence staining of inducible nitric oxide synthase (iNOS, a hallmark of pro‐inflammatory activation) in LPS‐stimulated BV2 microglial cells treated with Ctrl (LPS only), UC‐EVs, TFF‐EVs or TFF‐SEC‐EVs (scale bar: 50 µm). (F) Quantitative analysis of the relative mean fluorescence intensity of iNOS. (G, H) Secretion levels of the pro‐inflammatory cytokines (G) TNF‐α and (H) IL‐6 in LPS‐stimulated BV2 cells following treatment with the indicated EV preparations. Data are presented as mean ± SD (*n* = 3 independent experiments).

To further evaluate the functional relevance of the optimized EV preparations, we assessed the biological activities of EVs isolated by UC, TFF and TFF‐mSEC. Given the well‐documented pro‐angiogenic and anti‐inflammatory properties of ADSC‐EVs, our functional characterization encompassed three key assays: tube formation and scratch wound healing to evaluate pro‐angiogenic and migratory capacities, and suppression of LPS‐induced inflammation to assess anti‐inflammatory potential. As shown in Figure 6A, HUVECs co‐incubated with EVs for 4 h formed tubular structures, whereas untreated cells (Blank) displayed only sparse, fragmented networks. Notably, TFF‐mSEC‐EVs induced the most extensive and elongated tubular networks. Quantitative analysis confirmed that TFF‐mSEC‐EVs significantly enhanced angiogenesis compared to UC‐EVs, as evidenced by a greater total branch length (Figure 6B). In the scratch wound healing assay, EV‐treated groups exhibited improved migratory capacity compared to the untreated control; however, no statistically significant differences were observed among UC‐EVs, TFF‐EVs and TFF‐mSEC‐EVs (Figure 6C,D).

For the anti‐inflammatory assessment, BV2 microglial cells were stimulated with LPS. Immunofluorescence analysis revealed that LPS stimulation markedly increased the expression of inducible nitric oxide synthase (iNOS), a hallmark of pro‐inflammatory activation (Figure 6E). EV treatment showed a trend toward reduced iNOS expression; however, these changes did not reach statistical significance among different EV groups (Figure 6F). Similarly, ELISA results indicated a decreasing trend in the secretion of pro‐inflammatory cytokines TNF‐α and IL‐6 following EV treatment, but no significant differences were observed among the EV groups (Figure 6G,H). Together, these data demonstrate that EVs obtained from all three isolation methods retain fundamental biological activity. Notably, the TFF‐mSEC strategy leads to a selective enhancement in pro‐angiogenic capacity, whereas it shows no significant effect on migratory or anti‐inflammatory outcomes under the current experimental conditions. These findings suggest that the advantage of the cascaded purification workflow may be function‐specific rather than universally applicable across all biological readouts, while still supporting its ability to preserve EV functionality.

We next evaluated the technical universality of the LFCA platform using EVs from an alternative cell source: human UC‐MSCs cultured in a 3D microenvironment. The cluster structures of UC‐MSC‐EVs isolated via UC, TFF and TFF‐mSEC were analyzed using the LFCA platform, with TEM serving as a reference. As shown in Figure S18, EVs isolated by both UC and TFF/mSEC exhibited low proportions of Cluster A and clean backgrounds in TEM images, indicating high purity. To further assess EV purity in accordance with the MISEV guidelines and to provide orthogonal experimental validation beyond size‐ and morphology‐based analyses, we performed Western blot assays on EVs isolated from both ADSCs and UC‐MSCs using different methods (Figure S19). The results revealed consistent expression of the transmembrane EV marker CD81 and the cytosolic protein marker HSP70, while no detectable signal of the negative marker Calnexin. Notably, EVs isolated by TFF, which demonstrated relatively lower purity based on our LFCA platform, showed CD81 positivity but exhibited absent or markedly reduced levels of HSP70, indicating the presence of non‐EV components and confirming compromised purity. Together, these data provide direct molecular evidence of differences in EV purity among isolation strategies, corroborating the classification results derived from the LFCA platform. This orthogonal validation strengthens the purity assessment and underscores the broad applicability of the LFCA platform across diverse cellular sources and isolation methods.

## 4 Discussion

The present study addresses critical challenges in the characterization and production of therapeutic EVs by introducing the LFCA platform, which integrates nFCM for particle size distribution analysis with advanced clustering algorithms. This innovative approach enables rapid, precise, and quantitative EV analysis, significantly enhancing the ability to characterize EV size distribution, yield and purity. Leveraging the platform's high‐throughput capabilities, we systematically optimized the cascaded production workflow for ADSC‐derived EVs, from cell culture to final purification, ensuring both high yield and quality.

The LFCA platform's ability to resolve complex nanoparticle populations within 5 min using only 20 µL of samples represents a significant improvement over traditional methods, which often struggle to distinguish EVs from co‐isolated contaminants. Utilizing GMM and EM algorithms, the platform automatically differentiates EV subpopulations from non‐EV particles without the need for fluorescent labelling or extensive sample preparation, thereby preserving the native state of EVs. For example, the platform revealed that a microcarrier‐based 3D culture system increased ADSC‐derived EV secretion by 30‐fold compared to traditional 2D cultures. Additionally, the platform guided the optimization of separation techniques, achieving approximately 90% purity while maintaining EV integrity through the integration of TFF and mSEC. Most importantly, functional analyses confirmed the superior therapeutic potency of EVs produced via this optimized workflow, which demonstrated significantly enhanced pro‐angiogenic and anti‐inflammatory capacities compared to those isolated by conventional methods.

By incorporating the LFCA platform into the ADSC‐derived EV workflow, we addressed key challenges in EV production, including scalability, reproducibility and quality control. The platform's real‐time feedback on EV composition and purity enabled iterative process optimization, ensuring consistent and high‐quality EV preparations suitable for clinical applications. Beyond ADSC‐derived EVs, the platform is broadly applicable to EVs from other cell types, further enhancing its impact in EV research and biomanufacturing. The preserved biological functionality of the final EV products, as evidenced by robust angiogenic and anti‐inflammatory activities, underscores the clinical relevance of our integrated approach. Beyond ADSC‐derived EVs, the platform is broadly applicable to EVs from other cell types, further enhancing its impact in EV research and biomanufacturing.

In summary, the LFCA platform represents a significant advancement in EV research and therapeutic development. Its rapid, precise, and quantitative analysis capabilities address key challenges in EV characterization and facilitate the establishment of more efficient and scalable production workflows. As the demand for EV‐based therapeutics grows, the LFCA platform has the potential to play a key role in advancing both basic research and clinical applications, ultimately improving patient outcomes and fostering progress in regenerative medicine.

## Author Contributions

**Jing Zhou**: conceptualization, investigation, writing – original draft, methodology, validation, writing – review and editing, software, formal analysis, data curation, **Ping Chen**: methodology, investigation, formal analysis, validation, **Xin Chen**: investigation, methodology, formal analysis, **Xu Xiao**: investigation, methodology, **Haonan Di**: investigation, methodology, **Yunyun Hu**: investigation, methodology, **Yarong Zhen**: resources, writing – review and editing, **Xiaomei Yan**: conceptualization, funding acquisition, writing – review and editing, project administration, supervision, resources

## Conflicts of Interest

X.Y. discloses competing financial interests as a co‐founder and shareholder of NanoFCM Inc. (Xiamen, China), a company dedicated to the commercialization of nano‐flow cytometry (nFCM) technology. ORCID Xiaomei Yan: 0000‐0002‐7482‐6863.

## Supporting information




**Supporting Information**: jev270333‐sup‐0001‐SuppMat.docx

## Data Availability

The data that support the findings of this study are available from the corresponding author upon reasonable request.
