# Supplementary material for: Label‐Free Clustering Analysis Platform Drives Cascaded Workflow for Scalable Production of Therapeutic Extracellular Vesicles
Source: J Extracell Vesicles. 2026 Jun 23;15(6):e70333. doi: 10.1002/jev2.70333 (PMC13291197; doi:10.1002/jev2.70333)
Supplement: Supplementary file 1 — Supporting Information: jev270333‐sup‐0001‐SuppMat.docx [file JEV2-15-e70333-s001.docx]

**Supplementary Information (SI)**

**Label-Free Clustering Analysis Platform Drives Cascaded Workflow for Scalable Production of Therapeutic Extracellular Vesicles**

Jing Zhou^1^, Ping Chen^2^, Xin Chen^2^, Xu Xiao^2^, Haonan Di^2^, Yunyun Hu^2^, Yarong Zhen^3*^, Xiaomei Yan^1, 2*^

^1^ Discipline of Intelligent Instrument and Equipment, College of Chemistry and Chemical Engineering, Xiamen University, Xiamen 361005 Fujian, China

^2^ Department of Chemical Biology, MOE Key Laboratory of Spectrochemical Analysis & Instrumentation, Key Laboratory for Chemical Biology of Fujian Province, State Key Laboratory of Physical Chemistry of Solid Surfaces, College of Chemistry and Chemical Engineering, Xiamen University, Xiamen 361005 Fujian, China

^3^ Department of Plastic Surgery, Zhongshan Hospital of Xiamen University, School of Medicine, Xiamen University, Xiamen, 361004 Fujian, China

*Corresponding author:

Xiaomei Yan, E-mail: [xmyan@xmu.edu.cn](mailto:xmyan@xmu.edu.cn);

Yarong Zhen, E-mail: 13860423006@163.com

**The PDF file includes:**

Materials and Methods

Supplementary Note

Supplementary Figures S1 to S19

Supplementary Table S1

Materials and Methods

Materials

Ultra-pure water (18.2 MΩ) was prepared using a Millipore Milli-Q water purification system (PURELAB Ultra FLC00006307, ELGA) and used for all solutions. Human adipose-derived mesenchymal stem cell (ADSC) differentiation media for adipogenic (HUXMD-90031), osteogenic (HUXMD-90021), and chondrogenic (HUXMD-90041) differentiation were purchased from Saiye Biotech.

The following antibodies were obtained from BD Biosciences: purified mouse IgG1, Anti-Human CD29 (555442), CD73 (555593), CD90 (555593), CD105 (555690), CD34 (550760), and Alexa Fluor™ 488 Anti-CD45 (567401). Mouse anti-human CD44 (338802) was purchased from Biolegend. Alexa Fluor™ 488 and 647 NHS ester reagents were obtained from Thermo Fisher Scientific.

AF488 and AF647-conjugated antibodies were prepared by reacting the carboxyl groups (COOH) of the antibodies with the NHS ester groups of Alexa Fluor 488 and 647 NHS ester, respectively. The reaction was performed in 1× phosphate-buffered saline (PBS) (20× PBS, Sangon Biotech Co., Ltd., B540627-0500) supplemented with 1/10 volume of 1 M NaHCO_3_ (Sinopharm, China).

Human umbilical vein endothelial cells (HUVECs) were purchased from Shanghai Anwei Biological Technology Co., Ltd. (Shanghai, China). BV2 cells were acquired from the National Experimental Cell Resource Sharing Platform (Beijing, China). Human umbilical cord mesenchymal stem cells (UC-MSCs) were generously provided by Dr. Dongyan Shen at the First Affiliated Hospital of Xiamen University.

Isolation of Adipose-Derived Mesenchymal Stem Cells (ADSCs)

Ethical approval for the collection of human surplus adipose tissue from non-obese patients undergoing liposuction for orthopedic purposes was obtained from the Ethics Committee of Zhongshan Hospital, Xiamen University (ID: XMZSYY-AF-SC-12-03). The adipose tissue was washed twice with 2% penicillin/streptomycin (P/S) in phosphate-buffered saline (PBS) to remove blood and tumescent fluid. Tissue digestion was performed using 0.1% collagenase type I (Gibco, 17100017) at 37°C for 1 hour. Adipose-derived mesenchymal stromal cells (ADSCs) were isolated from the digested tissue via centrifugation and subsequently seeded onto plastic culture dishes. The cells were cultured at 37°C in a 5% CO_2_ humidified atmosphere until reaching 70–80% confluence in a monolayer. The growth medium for cell expansion consisted of DMEM/F-12 (Gibco, C11330500BT) supplemented with 10% fetal bovine serum (FBS) (ExCell Bio, FSP500), 1% P/S, and 20 ng/mL human recombinant basic fibroblast growth factor (bFGF) (Novoprotein, C046).

Flow Cytometry Analysis of ADSCs

The surface marker expression of ADSCs was assessed by flow cytometry. Briefly, 100 μL of passage 5 ADSCs (5×10⁶ cells/mL) was mixed with 2 μL of AF488-conjugated antibodies against CD29, CD73, CD34, CD45, and an IgG1 κ isotope control, along with 2 μL of AF647-conjugated antibodies against CD44, CD90, CD105, and an IgG1 κ isotope control. The mixtures were incubated at 37°C in the dark for 30 minutes. After incubation, the ADSCs were washed twice with 1% BSA-PBS by centrifuging at 800 ×g for 5 minutes at 4°C. The pellet was resuspended in 200 μL of PBS and analyzed using a CytoFlex flow cytometer (Beckman Coulter, Inc.). Data were analyzed using FlowJo software.

Differentiation of ADSCs

The ability of ADSCs to differentiate into adipocytes, osteoblasts, and chondroblasts was evaluated following the manufacturer's protocols for the reagent kits. For adipogenesis and osteogenesis differentiation, 2 × 10^5^ ADSCs were seeded onto a 6-well plate. Upon reaching 80% confluency, the medium was replaced every 3 days using an adipogenesis differentiation kit (Saiye Biotech, HUXMD-90031) and an osteogenesis differentiation kit (Saiye Biotech, HUXMD-90021). After 4 weeks of differentiation, samples were evaluated by Oil Red O staining for lipid droplets and Alizarin Red staining for calcified deposits. For chondrogenic differentiation, 4 × 10⁵ ADSCs were placed in a 15 mL centrifuge tube, washed twice with chondrogenic basal media supplemented with Supplement I, and resuspended in chondrogenic complete media (Saiye Biotech, HUXMD-90041). The cell suspension was centrifuged at 150 ×g for 5 minutes to promote cell aggregate formation and cultured at 37°C in 5% CO₂ until aggregates formed. The media was replaced every 3 days. After 4 weeks, the chondrogenic aggregates were fixed with paraformaldehyde, embedded in paraffin, sectioned, and stained with Alcian Blue.

Ultracentrifugation (UC)

For EV isolation and purification, residual cells were removed by centrifugation at 800 ×g for 5 minutes, followed by 3000 ×g for 20 minutes to eliminate cell debris. The precleared conditioned medium (CM) was centrifuged at 100,000 ×g at 4°C for 120 minutes using a Beckman Coulter XE-90K Ultracentrifuge equipped with an SW32 Ti rotor to pellet EVs. The supernatant was carefully removed, and the crude ADSC-EV pellet was resuspended in 12 mL ice-cold PBS and pooled. A second round of ultracentrifugation was performed using an SW41 Ti rotor at 100,000 ×g at 4°C for 120 minutes. The resulting pellets were resuspended in 50 µL PBS per 50 mL CM.

Triton X-100 Lysis

For EV lysis, 45 μL of EV sample (approximately 2 × 10¹⁰ particles/mL) was mixed with 5 μL of 10% Triton X-100 (Sigma-Aldrich, SL09531). After 30 minutes of incubation at 37°C, the sample was diluted 20–100 times and analyzed using nFCM.

BCA Protein Assay

ADSC-EVs were lysed with RIPA Lysis Buffer (Solarbio, R0020). Protein concentrations were quantified using the BCA Protein Assay Kit (Sangon Biotechnology Co., Ltd., C503021-0500) according to the manufacturer’s instructions.

Transmission Electron Microscopy (TEM)

A 5 μL aliquot of EV preparations (approximately 1 × 10¹¹ particles/mL) was applied to a glow-discharged formvar-carbon-coated grid (Zhongjing Scientific Instrument Technology Co., Ltd., Beijing, China) and allowed to settle for 3 minutes. The grid was rinsed with 5 μL of 2% phosphotungstic acid solution, followed by negative staining with another 5 μL of 2% phosphotungstic acid solution for 30 seconds. After staining, excess solution was removed, leaving a thin water film. The grid was imaged using a Tecnai G2 Spirit Bio Twin transmission electron microscope operating at 120 kV.

Immunofluorescent Staining of EVs

For immunofluorescence staining, 50 μL of EV sample (approximately 1 × 10¹⁰ particles/mL) was mixed with 1 μL of AF488-conjugated antibodies against tetraspanins (CD9, CD63, CD81) and MSC surface markers (CD73, CD90, CD105, CD29), along with an IgG1 κ isotype control. The mixture was incubated at 37°C for 30 minutes. Unbound antibodies were removed by washing twice with 1 mL PBS via ultracentrifugation at 100,000 ×g for 17 minutes at 4°C using a Beckman Coulter Optima Max-XP ultracentrifuge. The pellet was resuspended in 50 μL PBS for analysis by nFCM.

Construction and Computational Fluid Dynamics (CFD) Simulation of the TFF Device

The TFF system was assembled from key components including a peristaltic pump, hollow fiber filter, pressure gauge, three-way valve, and multiple tanks (Table S1), interconnected by 3.2 mm ID silicone tubing. Within this circuit, the pump drives fluid movement, the pressure gauge provides system feedback, the valve directs flow, and the hollow fiber membrane enables EV filtration and enrichment. The system operates in four modes: washing-filtration, concentration, cleaning, and back-flushing. The washing-filtration mode removes particles smaller than the membrane pore size; the concentration mode concentrates larger particles; the cleaning mode removes accumulated particles; and the back-flushing mode clears particles embedded in the pores. The device design incorporates a cascading flow path for efficient nanoparticle filtration. CFD simulations were conducted using COMSOL Multiphysics, modeling fluid movement with the incompressible Navier-Stokes equations and particle movement with Newton’s second law. Simulations were performed on a refined physical mesh.

Hollow Filter Cartridge Regeneration

The hollow fiber filter was regenerated through routine and chemical cleaning. The filter was first cleaned with DI water at a flow rate of 50 mL/min, followed by incubation with 2% Triton X-100 for 30 minutes. The regeneration process adhered to the protocol outlined in Figure S13, with transmembrane pressure (TMP) maintained below 3 psi to prevent membrane damage. The filter was then filled with 0.1 M NaOH and stored at 4°C.

Chromatographic Packing

For column packing, a Long Gravity Affinity Chromatography Column (purchased from Taobao) was packed with Capto Core 700 (Cytiva, 17548101) and Q Sepharose Fast Flow (Cytiva, 17051010) resins. Each resin was thoroughly resuspended to avoid bubble formation. For multimodal size exclusion chromatography (mSEC), 6 mL of Capto Core 700 resin was loaded into the column. The term "multimodal" refers to the intrinsic property of this resin, which integrates two complementary separation mechanisms: size exclusion—separating particles based on hydrodynamic diameter—and secondary interactions (e.g., weak hydrophobic and ionic affinities)—refining separation by surface characteristics. This combination helps the resin to resolve EVs from co-isolated contaminants such as protein aggregates and lipoproteins, which often exhibit similar size ranges to EVs and are difficult to remove using conventional SEC. Importantly, this single-step multimodal approach is distinct from sequential chromatographic methods that employ multiple columns or steps. For anion-exchange chromatography (AEC), 6 mL of Q Sepharose Fast Flow resin was used. After packing, the upper bed support was placed on the resin bed to prevent disturbance and drying.

Chromatographic Column Equilibration

The SEC (qEV single 70 nm) and mSEC columns were equilibrated by passing 3 × bed volume of PBS (0.22 μm filtered, 25°C) through the column before sample loading. Similarly, AEC columns were equilibrated with 3 × bed volume of equilibration buffer (0.22 μm filtered, 25°C).

Western blotting

Protein concentrations of cell lysates and EV preparations were determined using a BCA protein assay kit (Sangon, C503021-0500). Samples were normalized and mixed with loading buffer to obtain 20 μg total protein per lane. Proteins were separated on 4–15% Tris-Glycine gels (BBI, C651104-0001) and transferred onto PVDF membranes (0.45 μm, Solarbio) using a Trans-Blot Turbo Transfer System (Bio-Rad). Membranes were blocked with 5% non-fat milk in TBST (Tris-buffered saline with 0.1% Tween-20; BBI, A600669-0250) for 30 min at room temperature, followed by overnight incubation at 4°C with primary antibodies against EV markers, including rabbit anti-human HSP70 antibody (Abcam, ab181606), CD81 polyclonal antibody (Bioworld, BS70675), rabbit anti-human Calnexin antibody (Epizyme Biotech, R013378), and β-actin rabbit monoclonal antibody (STARTER, S0B0005). After washing, membranes were incubated with HRP-conjugated goat anti-rabbit IgG secondary antibody (Proteintech, SA00001-2) for 30 min at room temperature. Protein bands were visualized using enhanced chemiluminescence reagent (ECL, Beyotime, P0018S) and imaged with an Amersham Imager 600 (GE Healthcare).

Supplementary Note

Mathematical Model Construction

Given an size distribution dataset $D=\left\{\left(x_{i},y_{i}|i=1,2,\cdots n\right)\right\}$, where $x_{i}$ represents the particle size feature value (unit: nm) and $y_{i}$ denotes the measured value corresponding to $x_{i}$, an automated analysis of the particle size distribution is performed using a Gaussian Mixture Model (GMM) with a variable number of Gaussian components. This allows for the parameterized expression of the probability density function given by Equation (1.1):

where:

- $K\in\left[K_{\min,}K_{\max}\right]$: Number of Gaussian components (Estimated based on local extrema);
- $\theta=\left\{\omega_{j},\mu_{j},\sigma_{j}^{2}|j=1,\cdots,K\right\}$: Parameter set for GMM fitting;
- : Gaussian probability density function;
- $\omega_{j}$: Mixing weight coefficient, satisfying $\left(\sum_{j=1}^{K} \omega_{j}\right)=1$;
- $\mu_{j}$: Mean of the $j\text{th}$ Gaussian component;
- $\sigma_{j}^{2}$: Variance of the $j\text{th}$ Gaussian component;

Among them, the GMM mean values reflect the typical particle sizes of EV subpopulations, variances characterize the homogeneity of the EV biosynthesis process, and mixing weights indicate the relative abundance of subpopulations.

Parameter Estimation and Optimization

The LFCA algorithm constructs an iterative optimization framework based on the Expectation-Maximization (EM) principle, consisting of four main steps.

1. Data Preprocessing

Raw measurement data are first normalized to eliminate dimensional differences that may affect model fitting. The normalization process given by Equation (1.2):

$$y_{i}^{norm}=\frac{y_{i}}{\sum y_{i}}$$

The normalized data are then used as input for the GMM model. The number of particles at each particle size is calculated to ensure computational accuracy and efficiency, with a base count $N=10000$. The calculation of the number of particles at each particle size is given by Equation (1.3):

$$y_{i}^{input}=N*y_{i}^{norm}$$

Further, the results obtained from the above equation serve as input for the LFCA algorithm, which is expressed in the following form: , where $x_{i}^{1}=x_{i}^{2}=...=x_{i}^{m}$, and *m* represents the number of particles with a size of $x_{i}$.

2 Initialization of GMM Parameters

The initial parameters for the GMM are set as follows: The means follow a uniform distribution: $u_{i}^{init}=\frac{i}{K}\cdot\left(\max(y^{input})-\min(y^{input})\right)$. Randomly assigned variance values in the range of 5 to 20: $\sigma_{i}^{init}=random(5,20)$. Weight coefficients set as the reciprocal of the number of Gaussian components to ensure their sum equals 1: $\omega_{i}^{init}=\frac{1}{K}$

3. EM Algorithm Iterative Optimization

Parameter estimation is achieved by iteratively maximizing the log-likelihood function, with the objective function given by Equation (1.4):

$$\begin{matrix} Q(\theta,\theta^{t})= \sum_{i} \sum_{j} \gamma_{j}^{(t)}\ln\frac{\omega_{j}}{\gamma_{j}^{(t)}\sqrt{\left(2\pi\right)^{d}\det\left(\Sigma_{j}\right)}} \\ \exp\left[-\frac{1}{2}\left(y_{i}^{input}-\mu_{j}\right)\Sigma_{j}^{-1}\left(y_{i}^{input}-\mu_{j}\right)\right] \end{matrix}$$

Simultaneously, the maximum likelihood probability is computed to determine the best-fitting parameters, as expressed in Equation (1.5):

$$\theta=\mathop{\mathrm{argmax}}\limits_{1\leq t\leq loop_{\text{num}}} (likelihood^{(t)})$$

The likelihood function is calculated using Equation (1.6):

$$likelihood^{(t)}=\sum_{j=1}^{K} \omega_{j}^{(t)}\frac{1}{\sqrt{2\pi\sigma_{j}^{(t)}}}\exp\left(-\frac{\left(y^{input}-\mu_{j}^{(t)}\right)^{2}}{2\sigma_{j}^{2(t)}}\right)$$

**(1) E-step (Expectation Calculation)**

The posterior probability is computed using Equation (1.7):

where $\gamma_{j}^{(t)}$represents the posterior probability that a particle with size $x_{i}$belongs to $j\text{th}$the Gaussian component at the $t\text{th}$iteration.

**(2) M-step (Maximization Update)**

The GMM parameters, including the weight coefficients, means and standard deviations are updated using Equation (1.8-1.10):

The iteration terminates when either the number of iterations $iter_{num}$ reaches the predefined maximum iteration count $loop_{num}$ or the convergence threshold $Th_{c}$ is met. The stopping criteria are defined using Equation (1.11):

$$\left\{\begin{matrix} iter_{num}\geq loop_{num} \\ \frac{\gamma^{(t+1)}-\gamma^{(t)}}{\gamma^{(t)}}<Th_{c} \end{matrix}\right.$$

where $loop_{num}$ is set to 1500 and $Th_{c}$ is set to 0.0001.

**(3) Goodness-of-Fit Evaluation**

The residual sum of squares (*RSS*) is used as a metric to assess the goodness of fit between the model and the original data. The calculation formula is given in Equation (1.12):

Where $y_{i}$ represents the original data points, and represents the model-fitted values. A lower *RSS* value indicates a better fit. By evaluating the goodness of fit under different *K* values, the optimal number of Gaussian components is selected.

**4. Result Output**

For each candidate number of components $K\in\left[K_{\min,}K_{\max}\right]$, the full EM fitting procedure is executed, generating the following outputs:

(1) The original particle size distribution histogram;

(2) The probability density curve of the mixture distribution .

**Supplementary Figures**

**
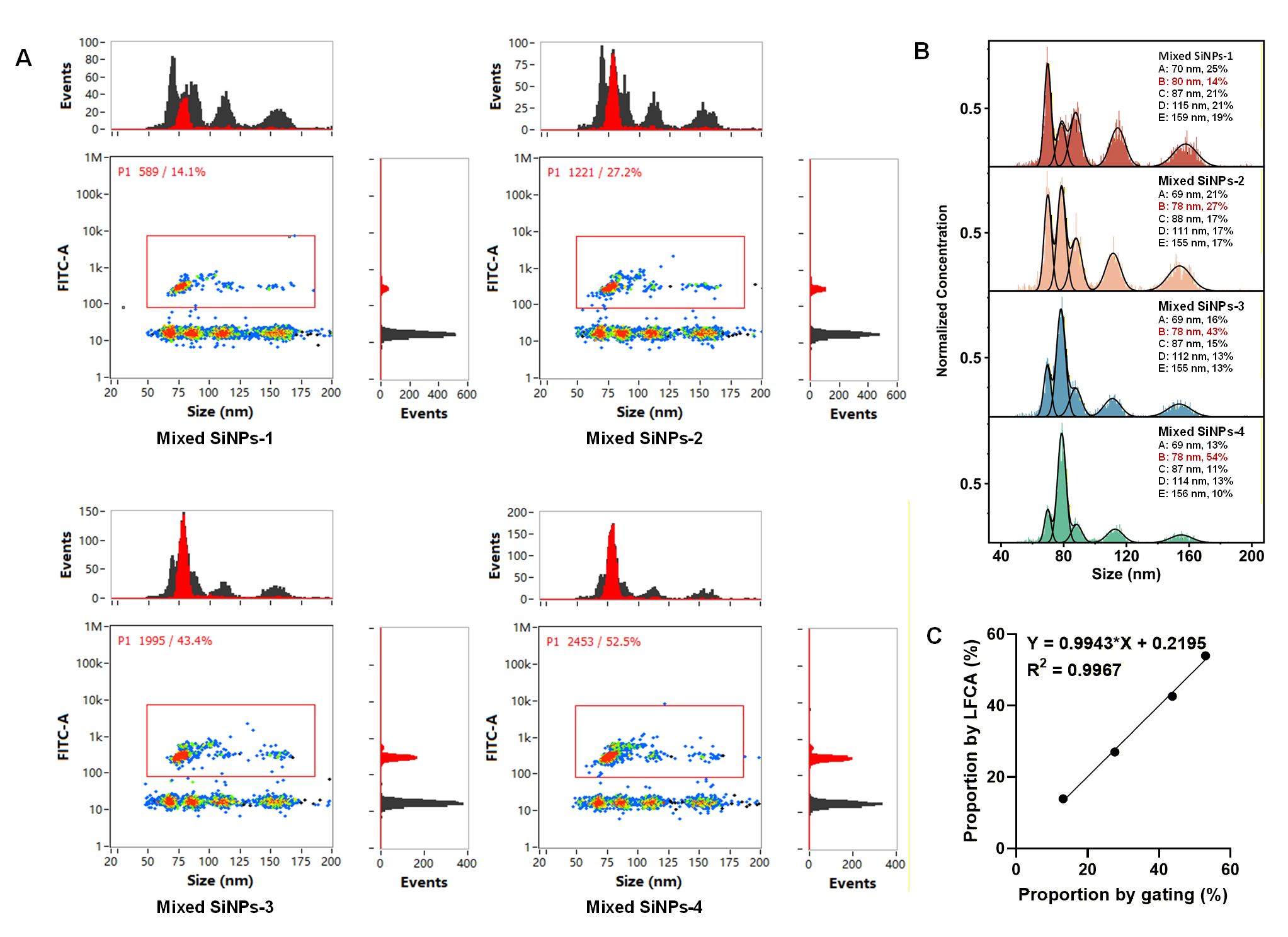
**

**Figure S1.** Validation of the LFCA platform for clustering analysis. (A) Gating analysis of 78 nm FL-SiNPs within mixed samples, showing varying proportions of these nanoparticles. The upper histogram illustrates the size distribution, while the right histogram displays the fluorescence intensity distribution of the mixed SiNPs. (B) Clustering analysis of mixed SiNPs samples performed using the LFCA platform. (C) Linear correlation between the proportions of 78 nm FL-SiNPs determined through gating analysis and those obtained via clustering analysis with the LFCA platform.


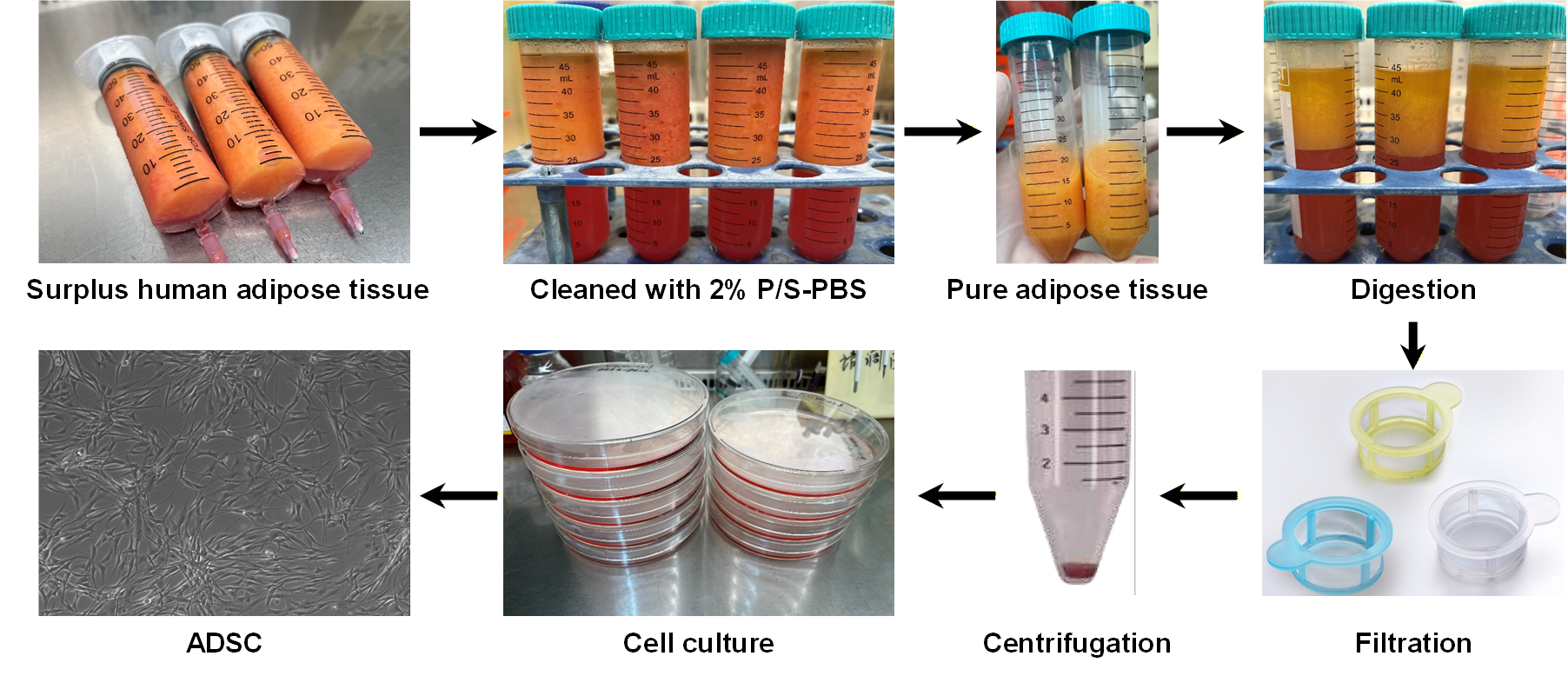


**Figure S2.** Schematic diagram of ADSC extraction workflow.

**
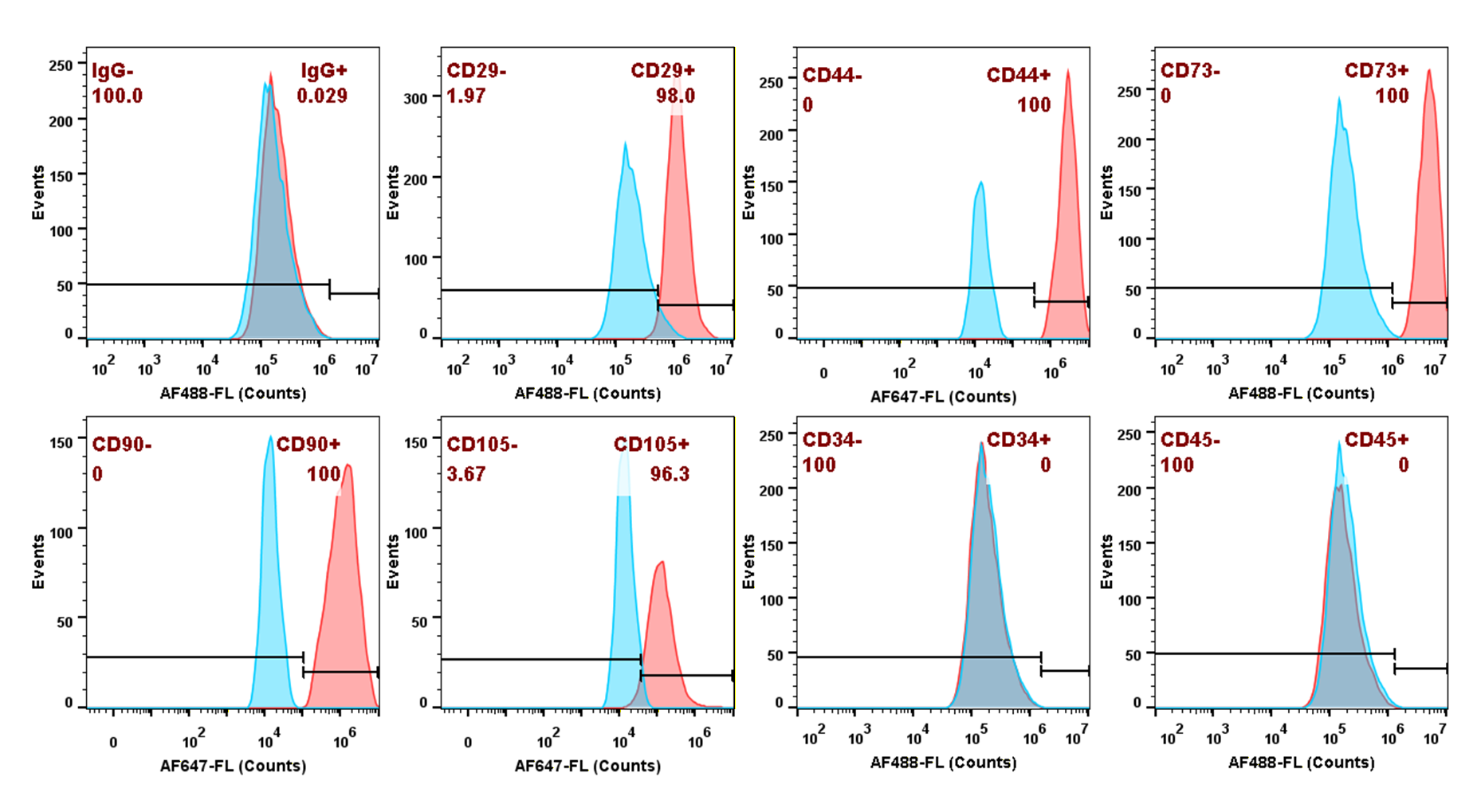
**

**Figure S3.** Flow cytometry results showing positive markers (CD29, CD44, CD73, CD90, CD105) and negative markers (CD34, CD45) on the surface of ADSCs.

**
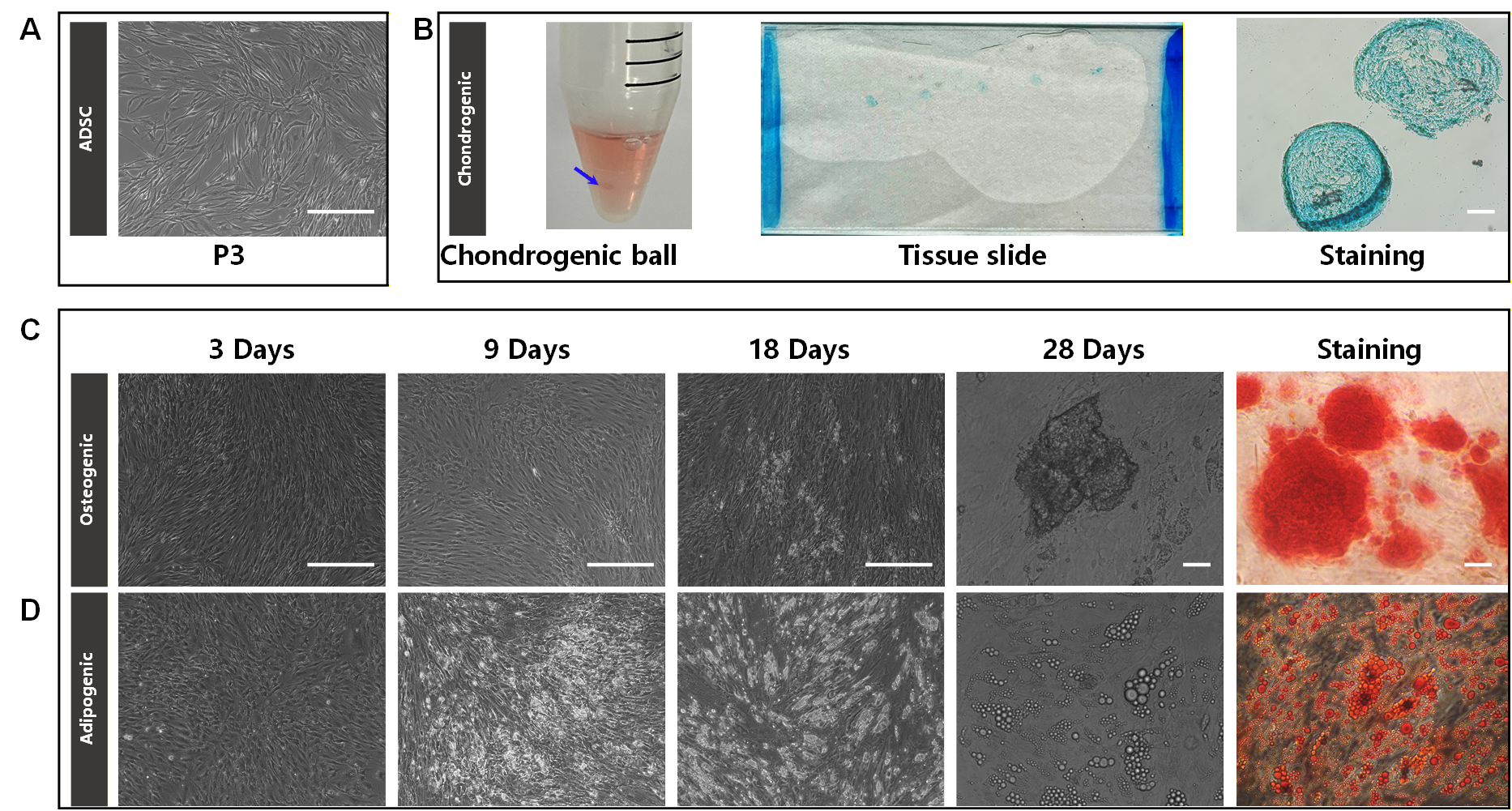
**

**Figure S4.** Optical microscopy images of ADSC trilineage differentiation results. Adipocytes, osteocytes, and chondrocytes were stained with Oil Red O, Alizarin Red, and Alcian Blue, respectively (scale bar: 100 μm).

**
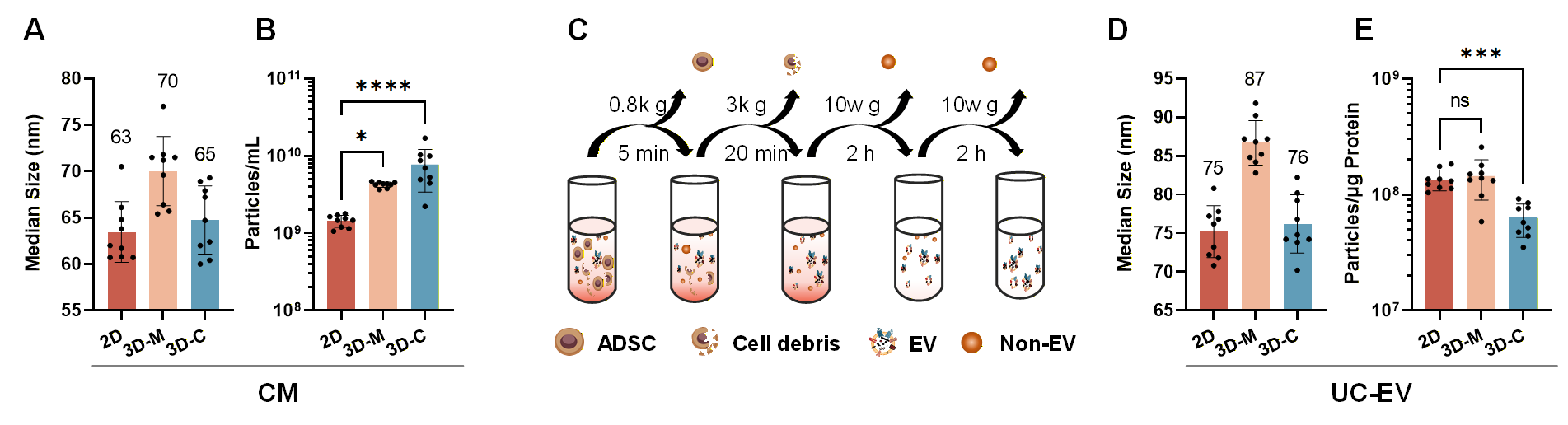
**

**Figure S5.** Characterization of nanoparticles in different culture systems by nFCM. Histograms of (A) median particle size and (B) concentration of nanoparticles in conditioned media (CM). (C) Experimental flowchart for UC-based EV extraction. (D) Median size and (E) particle-to-protein ratio graphs of UC-EV preparations. (n = 9; independent technical replicates, mean ± SD)

**
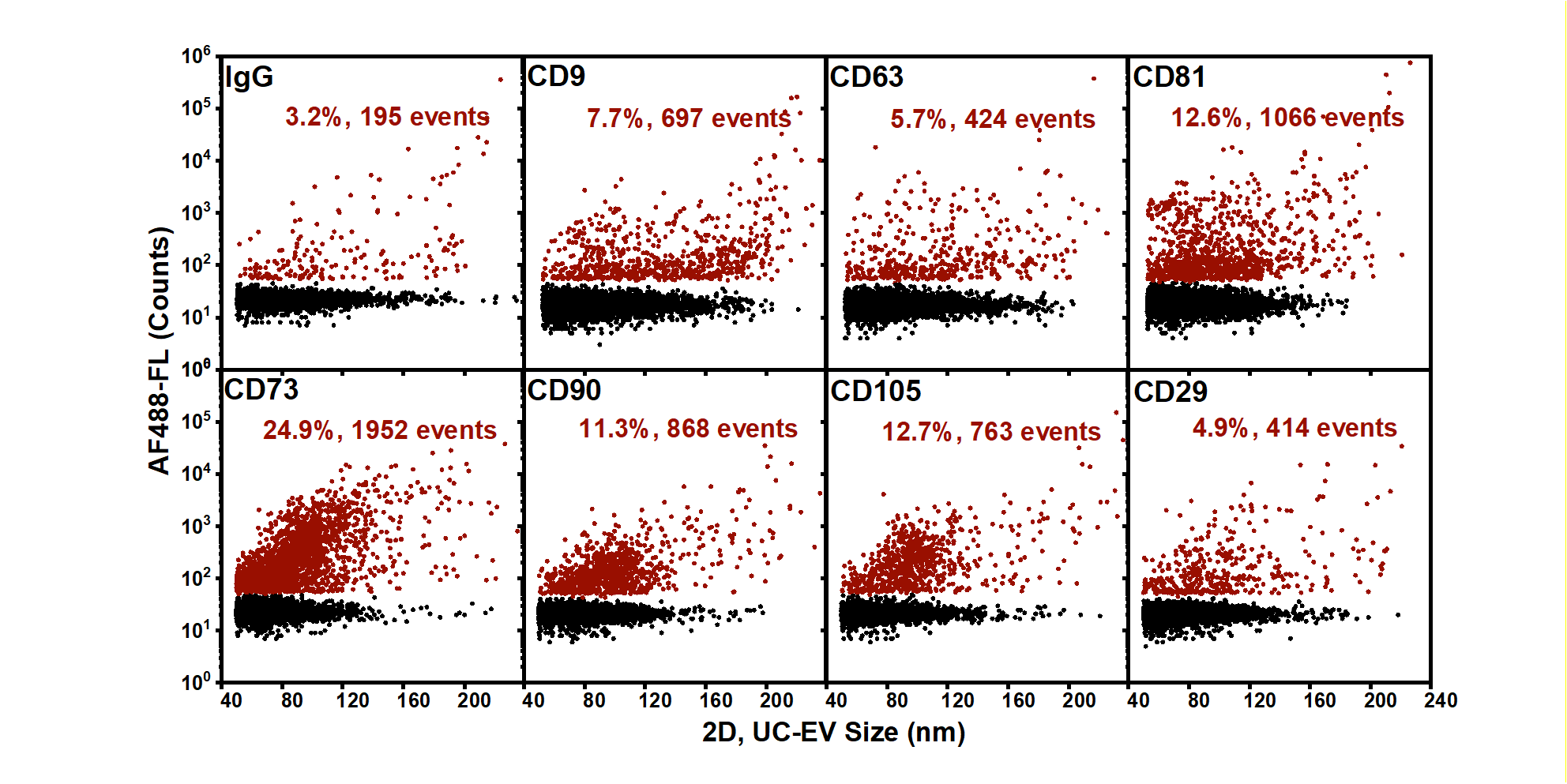
**

**Figure S6.** Immunophenotypic analysis of ADSC-EV preparations from 2D culture system isolated by UC. Bivariate dot-plots of AF488 fluorescence (FL) versus particle size for 2D UC-EVs labeled with antibodies against CD9, CD63, CD81, CD73, CD90, CD105, and CD29. Red dots represent protein-positive nanoparticles, and black dots represent protein-negative nanoparticles.

**
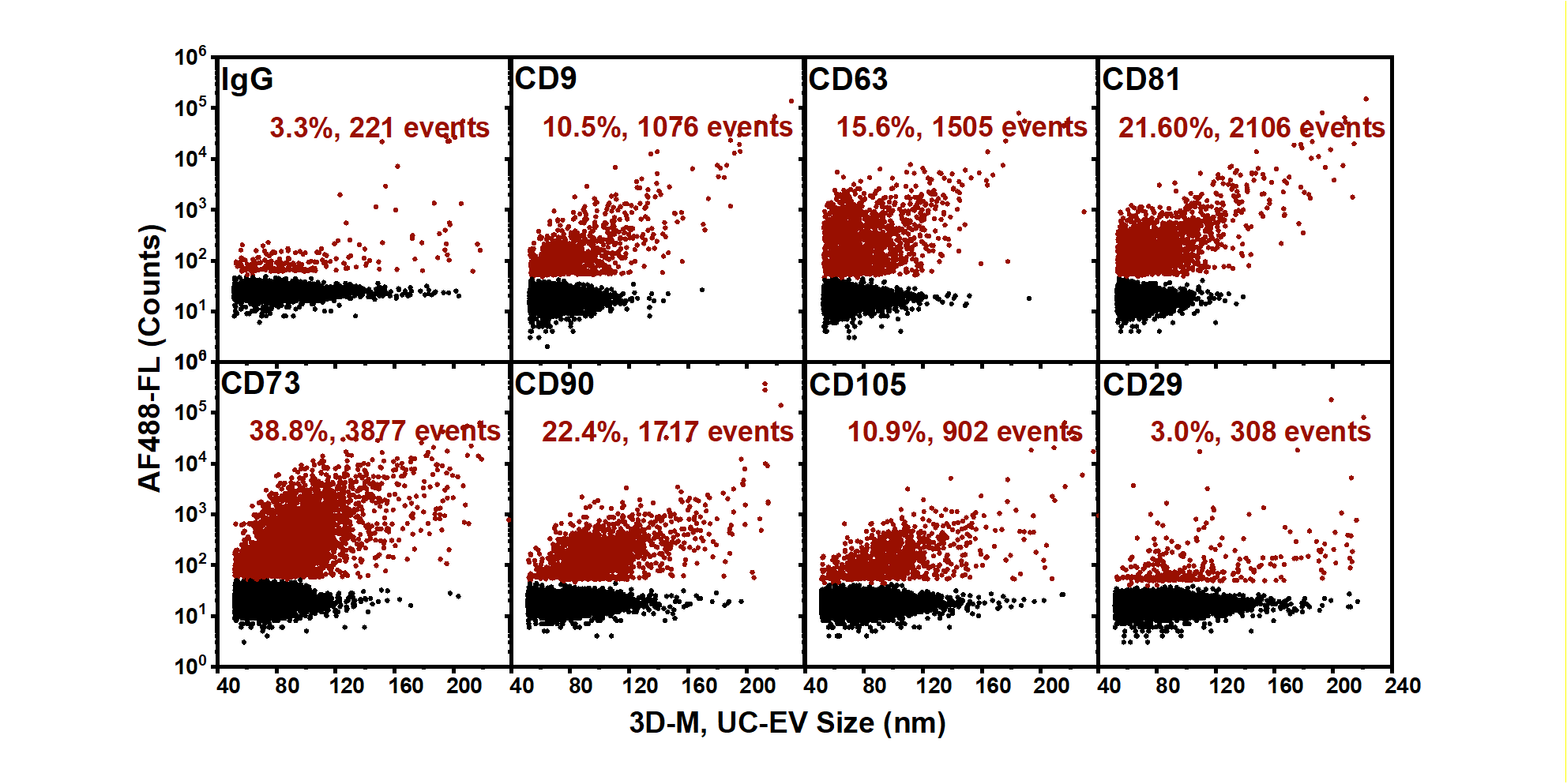
**

**Figure S7.** Immunophenotypic analysis of ADSC-EV preparations from 3D-M culture system isolated by UC. Bivariate dot-plots of AF488 fluorescence (FL) versus size for 3D-M UC-EVs labeled with antibodies against CD9, CD63, CD81, CD73, CD90, CD105, and CD29. Red dots represent protein-positive nanoparticles, and black dots represent protein-negative nanoparticles.

**
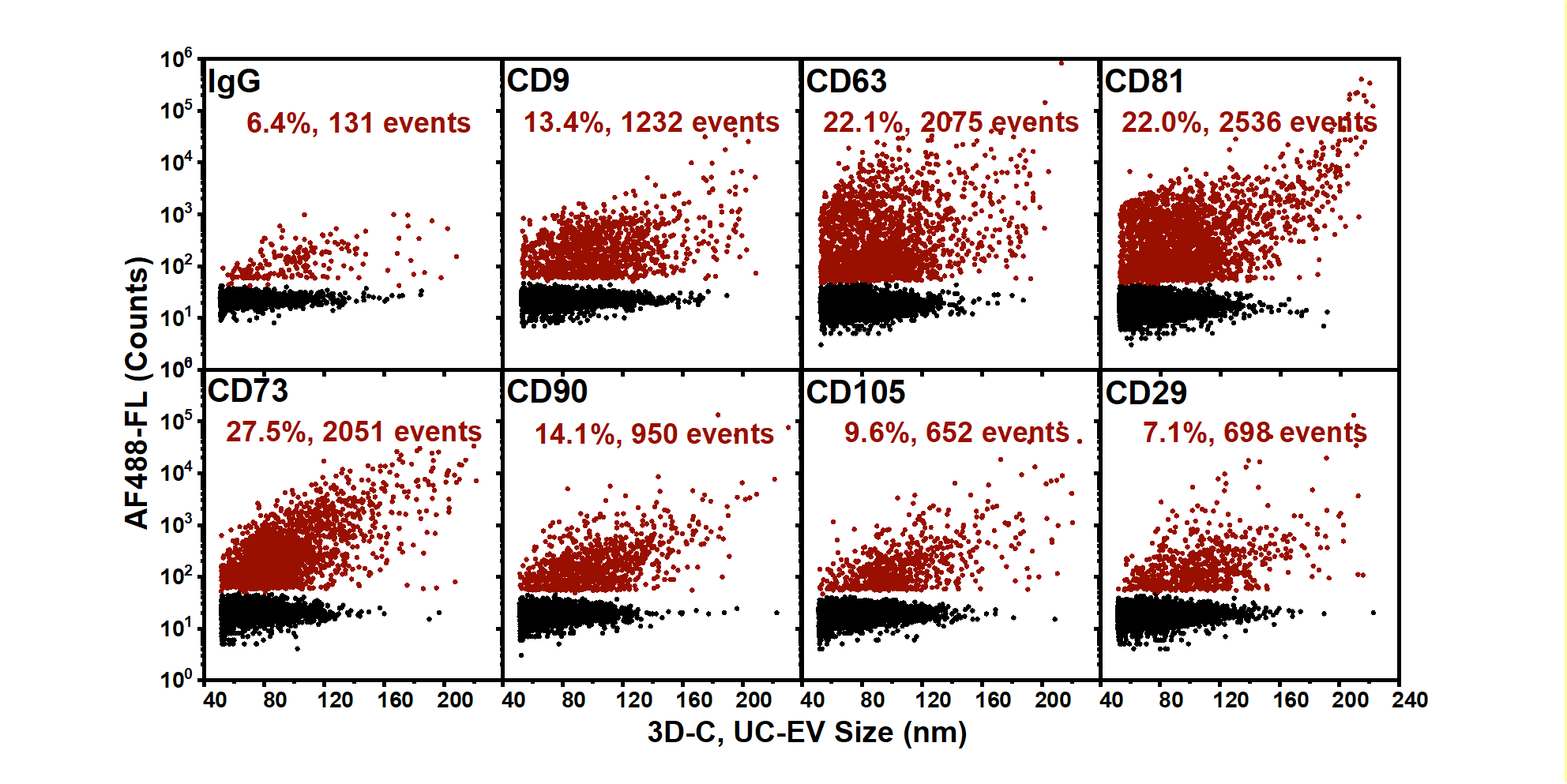
**

**Figure S8.** Immunophenotypic analysis of ADSC-EV preparations from 3D-C culture system isolated by UC. Bivariate dot-plots of AF488 fluorescence (FL) versus size for 3D-C UC-EVs labeled with antibodies against CD9, CD63, CD81, CD73, CD90, CD105, and CD29. Red dots represent protein-positive nanoparticles, and black dots represent protein-negative nanoparticles.

**
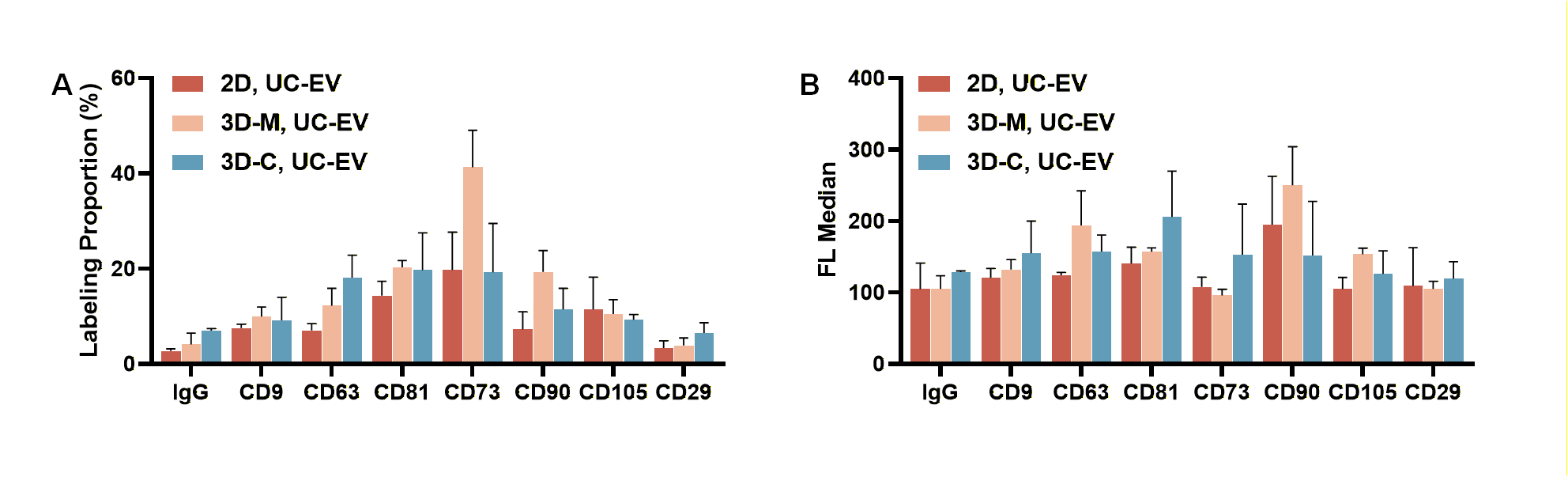
**

**Figure S9.** Immunophenotypic analysis of UC-EV preparations obtained in different culture systems by nFCM. Comparison of (F) the proportion and (G) median fluorescence intensity of protein-positive EVs in ADSC-EV preparations from different culture systems. (n = 3; independent technical replicates, mean ± SD).

**
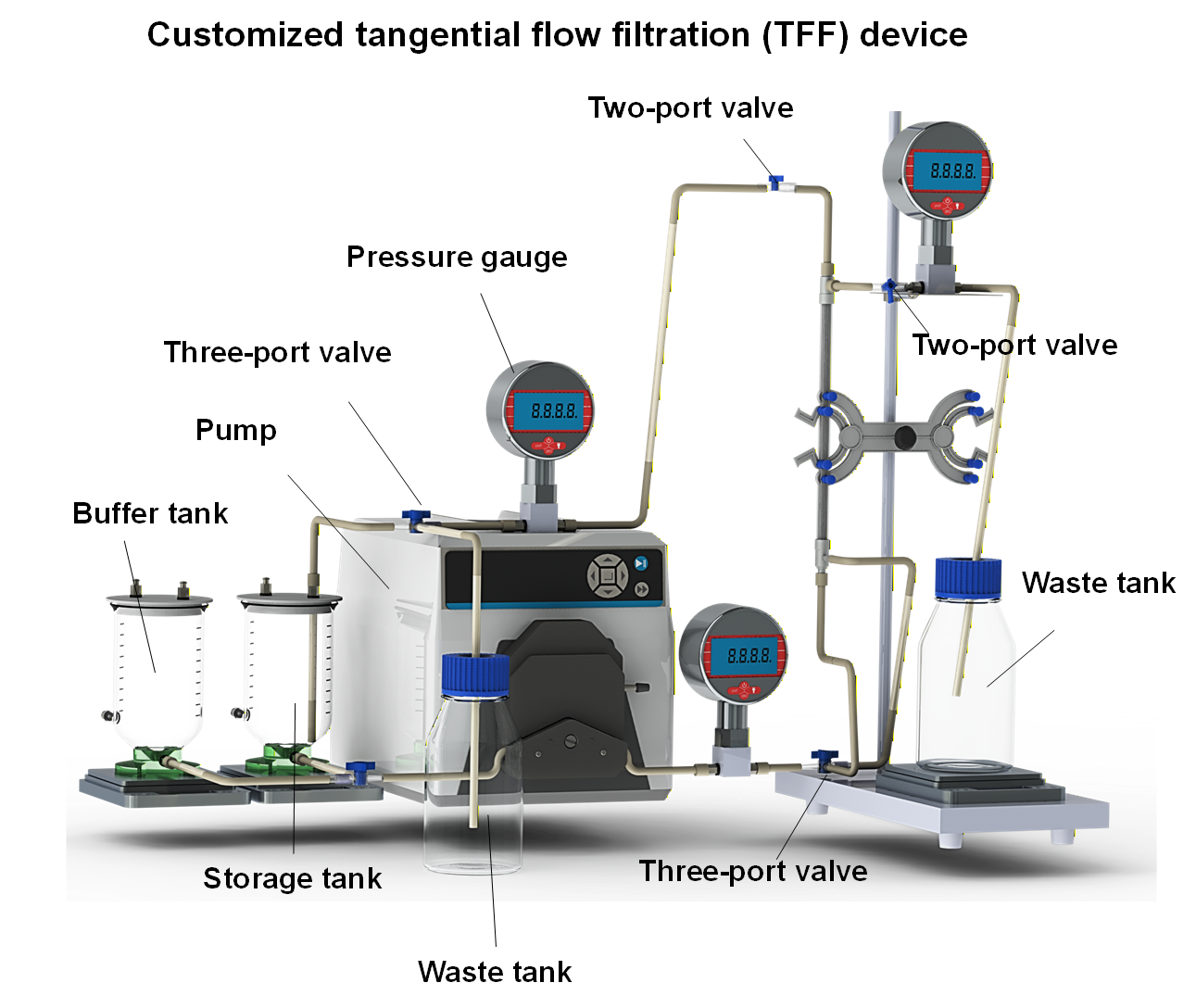
**

**Figure S10.** SolidWorks model diagram of the customized TFF device.

**
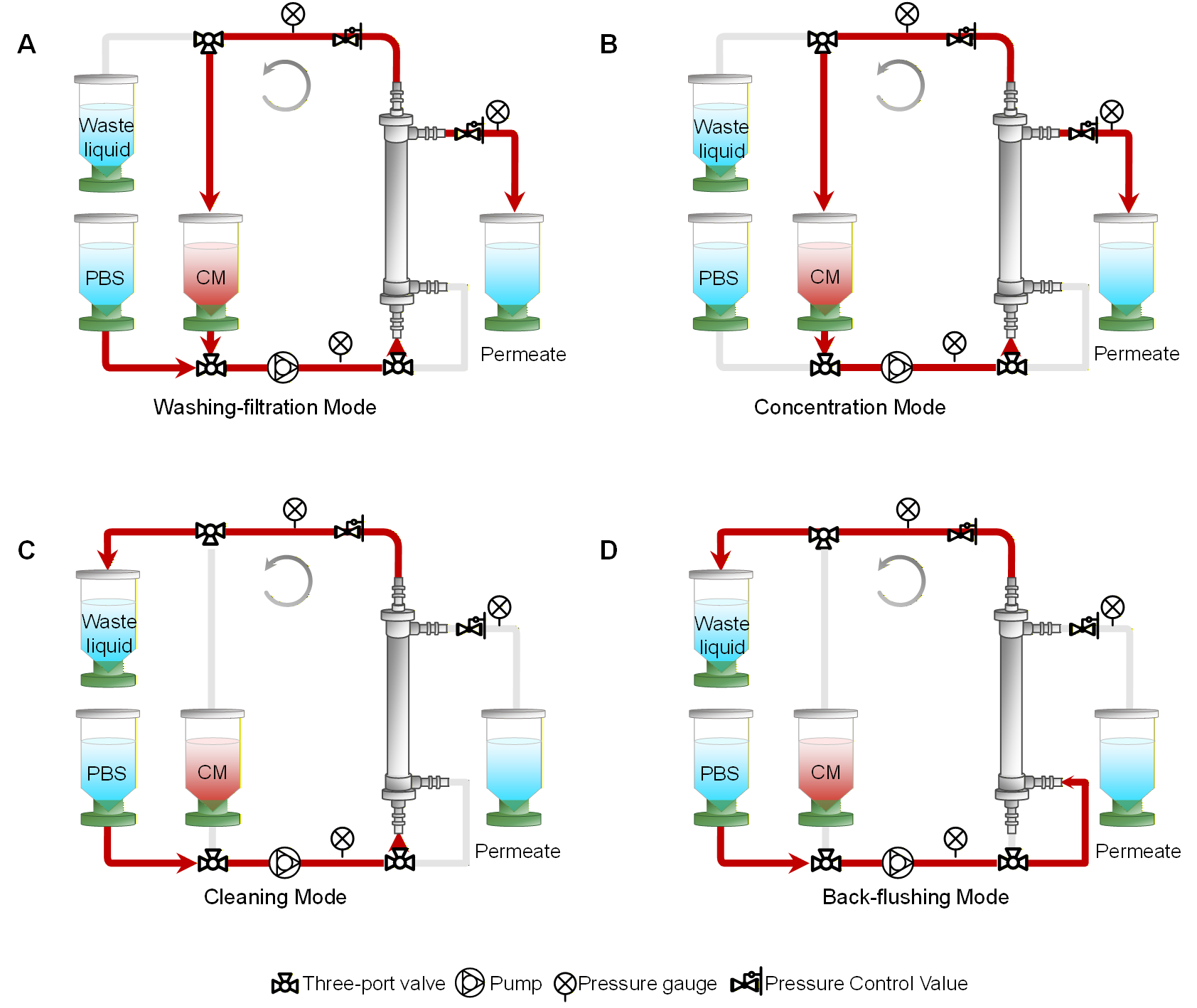
**

**Figure S11.** Flow path mode schematic of the TFF device. (A) Washing-filtration Mode, (B) Concentration Mode, (C) Cleaning Mode, and (D) Back-flushing Mode.

**
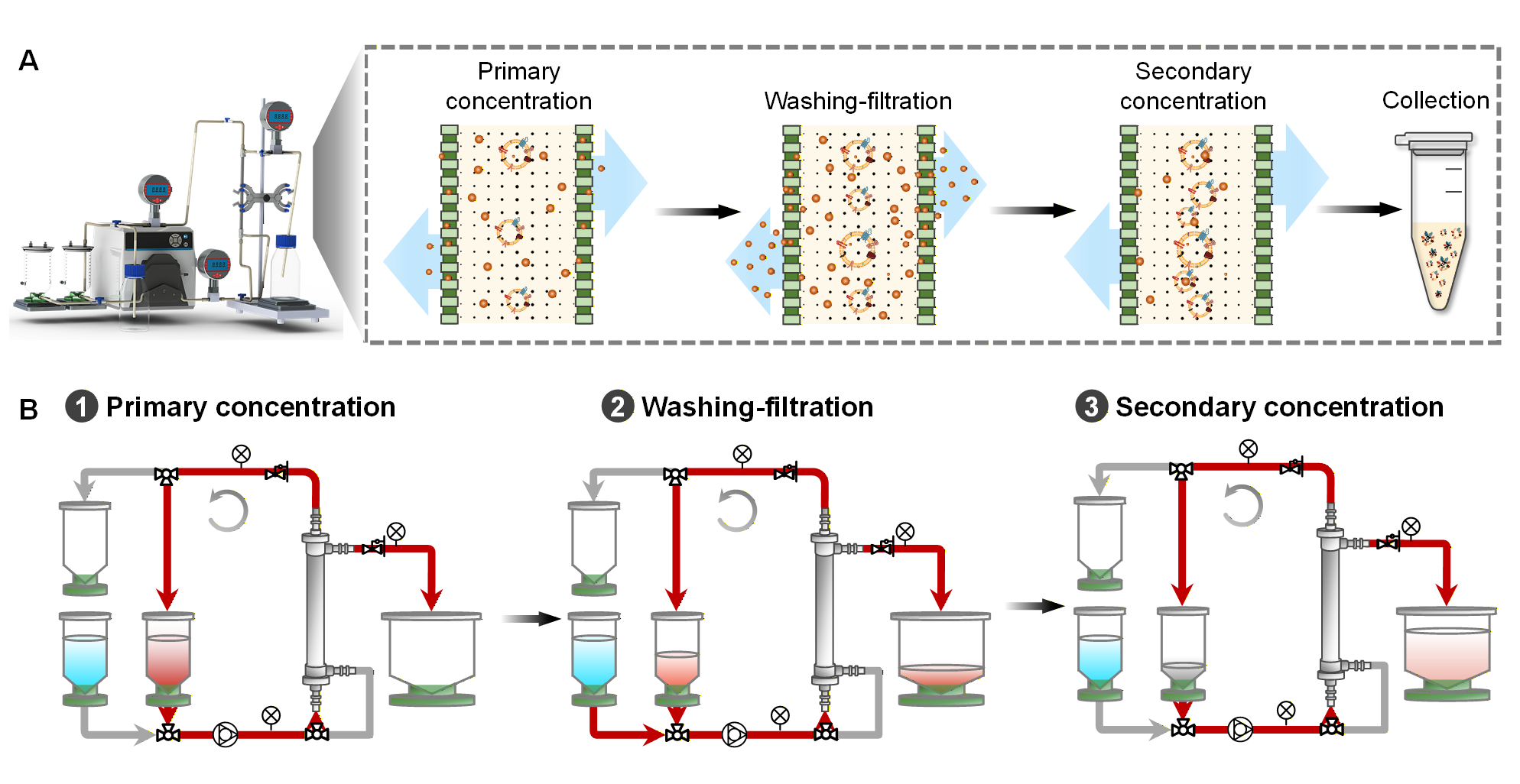
**

**Figure S12.** (A) Diagram and (B) Schematic of the TFF-based separation process. The process includes three main steps: Primary Concentration, Washing-filtration, and Secondary Concentration.

**
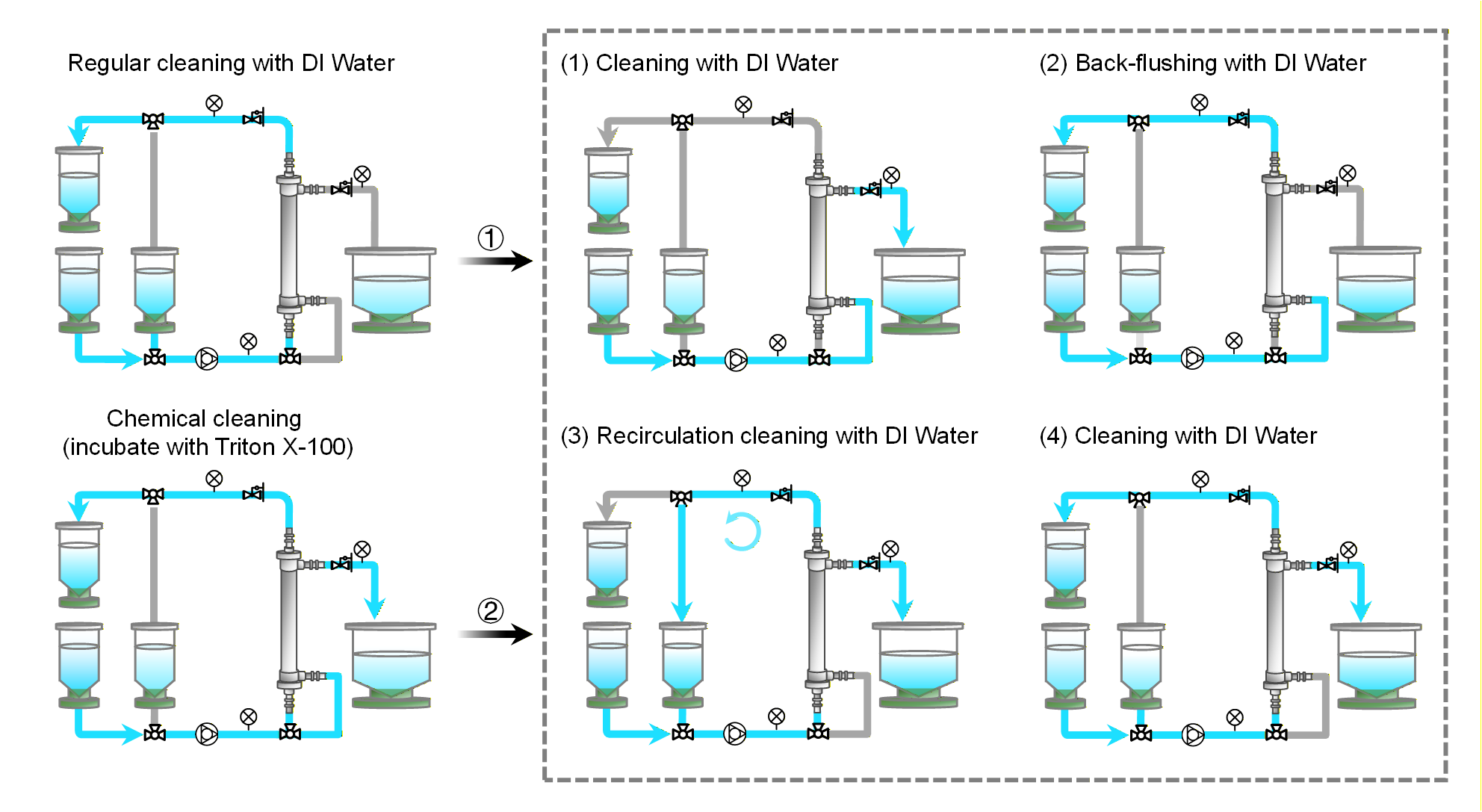
**

**Figure S13.** Regeneration flowchart of the hollow fiber filter cartridge. Regeneration steps include Regular Cleaning and Chemical Cleaning.

**
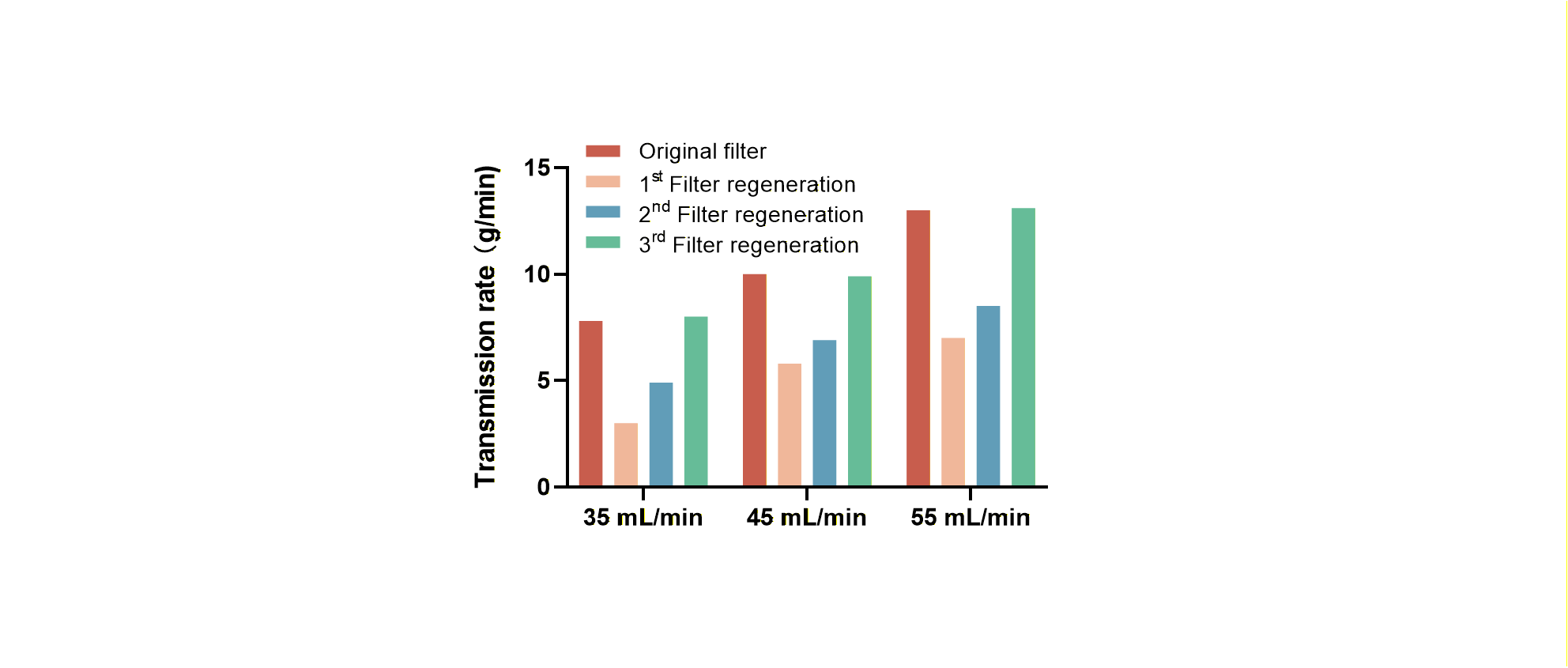
**

**Figure S14.** Comparison of water flux in hollow fiber membranes after 1^st^, 2^nd^, and 3^rd^ rounds of regeneration.

**
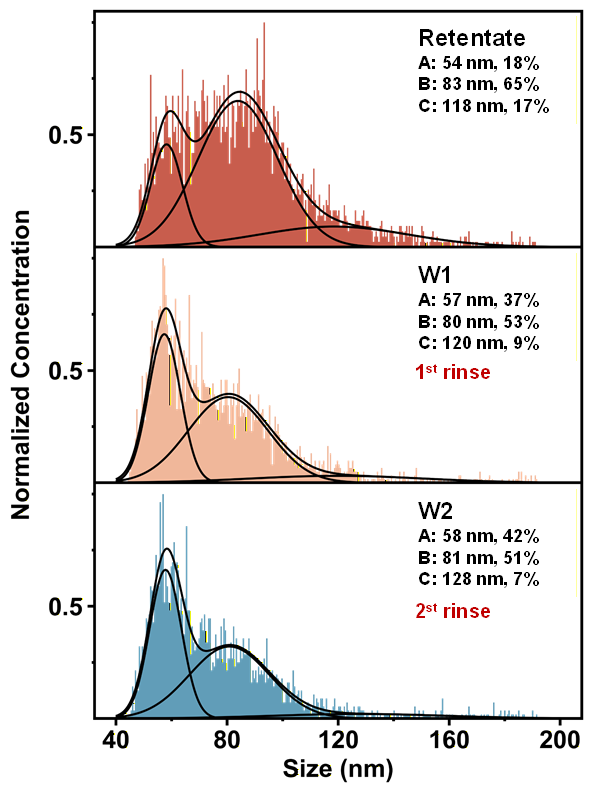
**

**Figure S15.** Clustering analysis results of retentates obtained from the initial Run and the 1^st^ and 2^nd^ rinses (W1 and W2) of the TFF device inner wall.

**
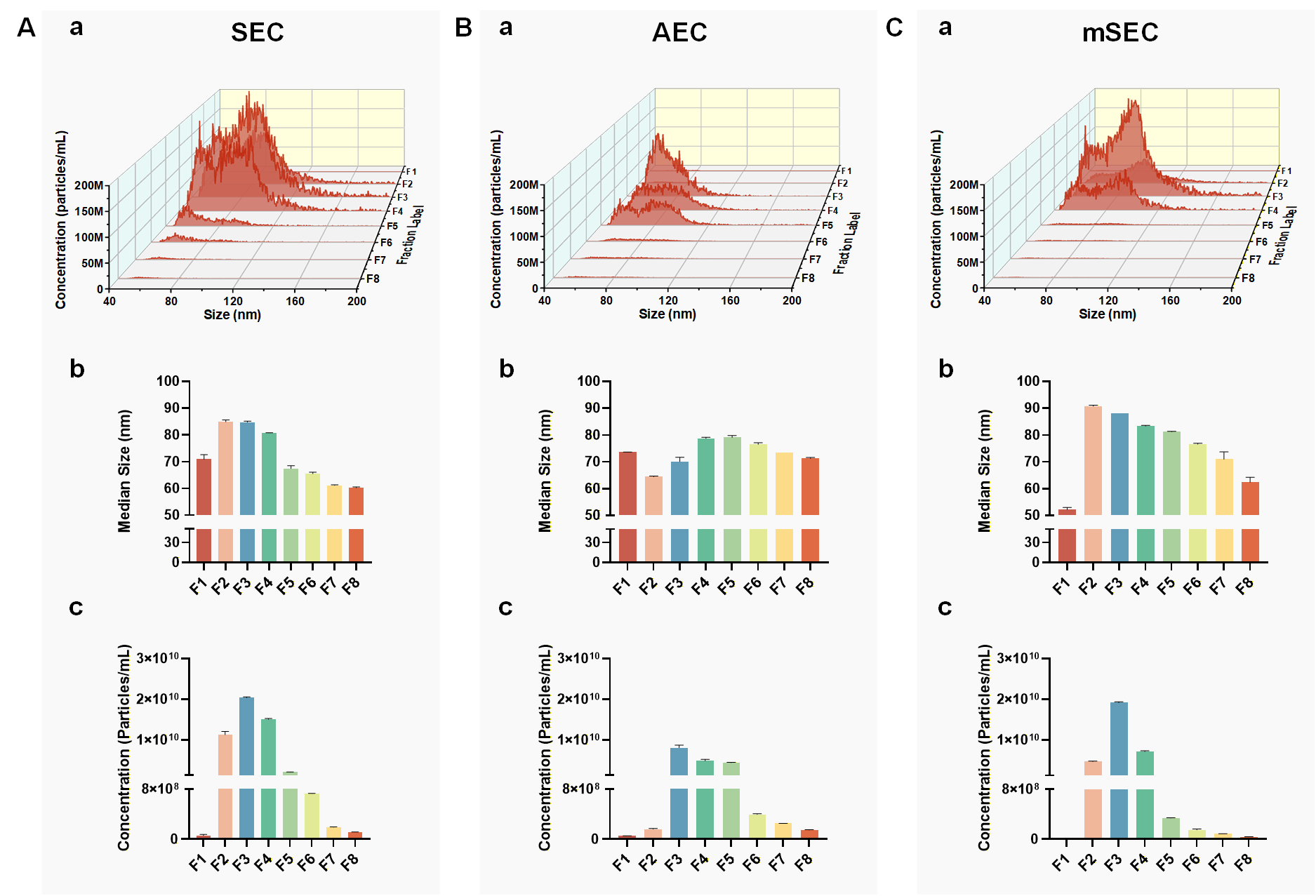
**

**Figure S16.** Characterization results of median sizes and concentrations for fractions F1-F8 obtained from SEC, AEC, and mSEC, as determined by nFCM. (a) Size distribution histogram, (b) median sizes, and (c) concentrations of F1-F8 fractions for (A) SEC, (B) AEC, and (C) mSEC. (n = 3; independent technical replicates, mean ± SD)

**
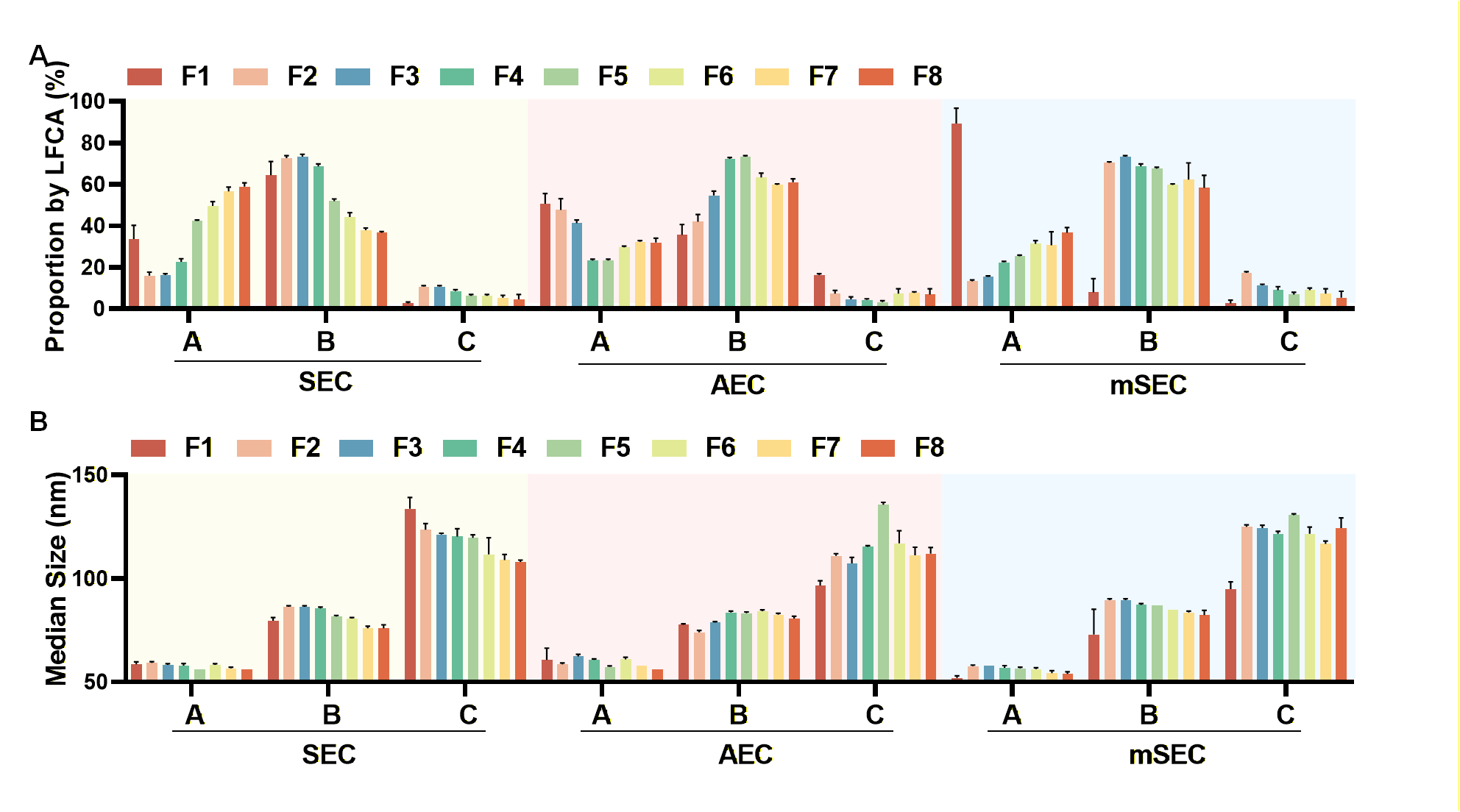
**

**Figure S17.** (A) Cluster analysis of clusters A, B, and C in fractions F1-F8 obtained from SEC, AEC, and mSEC. (B) Comparison of the proportions and median sizes of Clusters A, B, and C. (n = 3; independent technical replicates, mean ± SD).

**
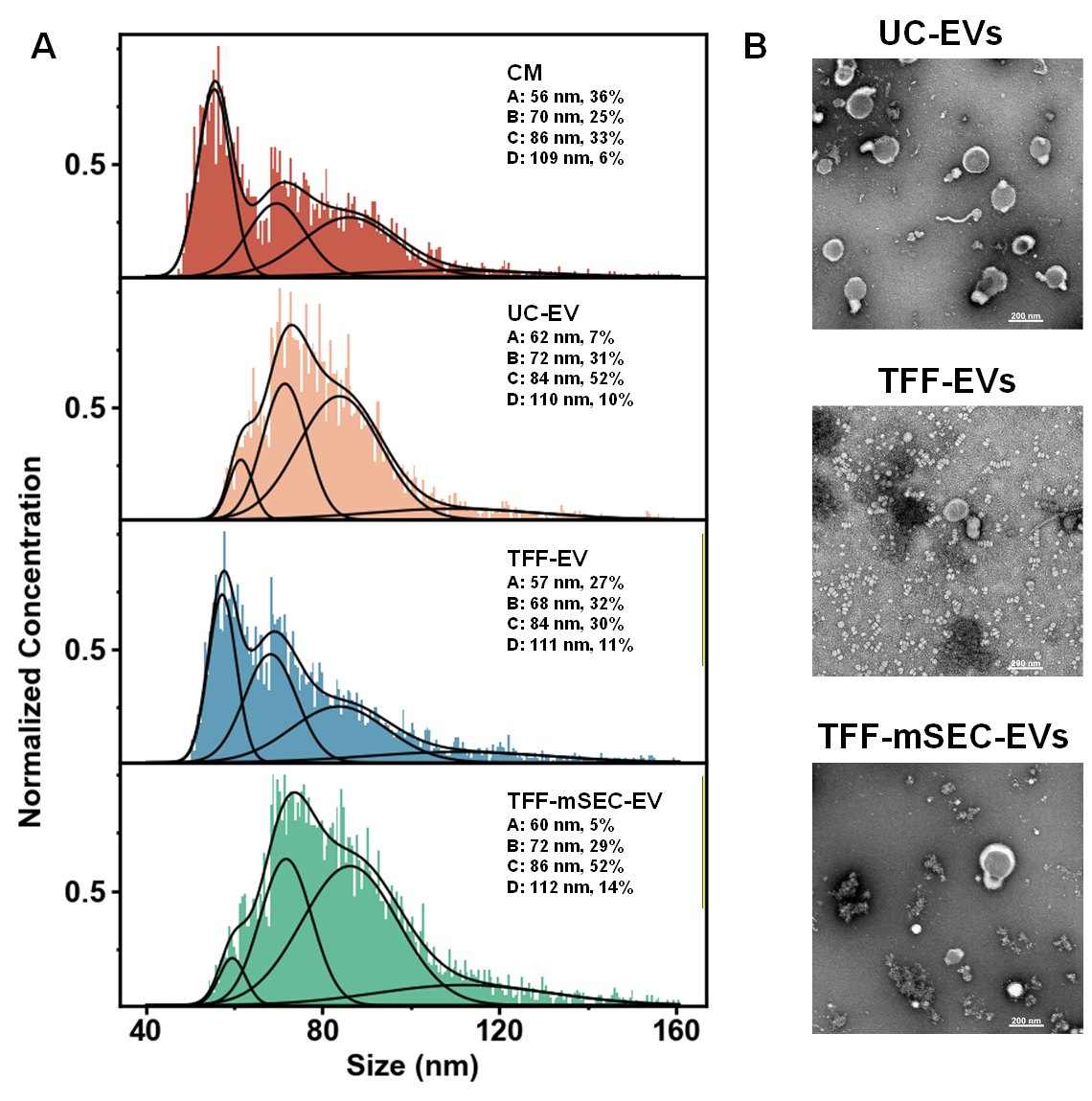
**

**Figure S18. Analysis of UC-MSC-derived EVs. (A) Clustering analysis of conditioned medium (CM) and EVs isolated by UC, TFF, and TFF-mSEC, showing the size distribution and proportion of distinct subpopulations (Clusters A–D). (B) Representative TEM images of the resulting EV preparations (scale bar: 200 nm).**

**
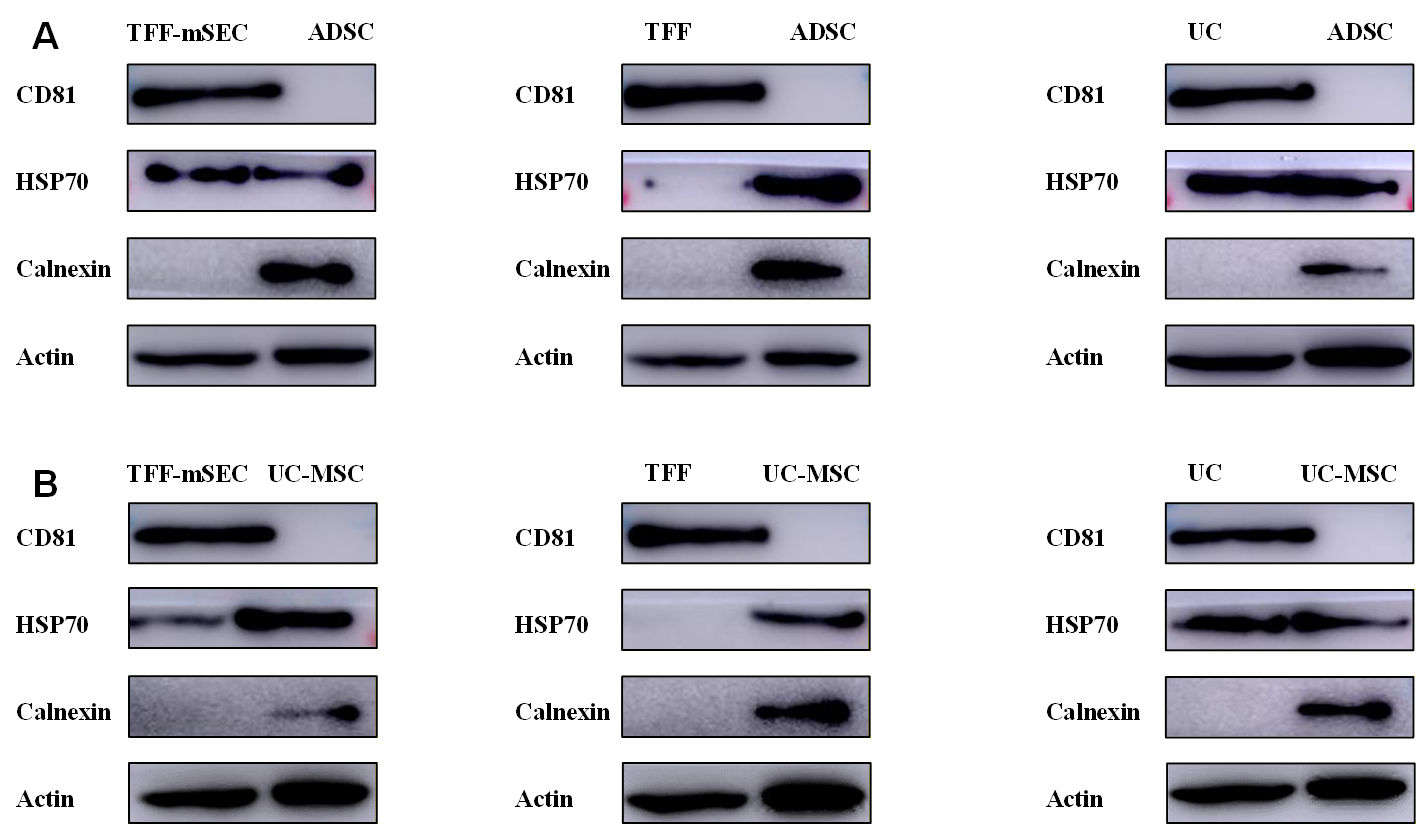
**

**Figure S19. Western blot validation of EV preparations for purity assessment. EVs isolated from ADSCs and UC-MSCs via ultracentrifugation (UC), tangential flow filtration (TFF), and the combined TFF‑multimodal size‑exclusion chromatography (TFF‑mSEC) workflow were analyzed for the expression of classic EV markers. CD81 and HSP70 served as positive markers, Calnexin as a negative (non‑EV) marker, and Actin as a loading control. EV preparations obtained by UC and TFF‑mSEC showed consistent expression of both CD81 and HSP70, with no detectable Calnexin signal. In contrast, TFF‑EVs retained CD81 positivity but exhibited markedly reduced or absent HSP70 expression, indicating a lower degree of purity, which aligns with the LFCA‑based classification.**

**Table S1.** Specifications of the custom-designed tangential flow filtration (TFF) system.

| **Component name** | **Manufacturer** | **Dimension** |
| --- | --- | --- |
| Peristaltic pump | Masterflex, model 07522-20 | – |
| Silicone tubing | Saint-Gobain BPT Rubber Hose | 3.2 mm × 6.4 mm (ID × OD) |
| Pressure gauge | Meacon instruments | M20 × 1.5, 0-0.6 mPa |
| Three-way valve | Custom-designed | M20 × M6 |
| Stainless steel adapter | Custom-designed | M6 × 1 mm |
| Electronic balance | Taobao | 10 kg, 0.01g readability |
| Hollow fiber filter | Repligen (D02-E500-05-N) | 20 cm length; mPES membrane; 500 kDa MWCO |
| Buffer/storage tank | Custom-designed | 750 mL |
| Waster liquid/permeate tank | Wenoote (Taobao) | 500 mL |
| Luer connectors | Dongshen Industries | 3.2 mm inner diameter |
| Three-way luer valve | MeiRui Medical | 3.2 mm inner diameter |
